# A Toaster in the Bathroom: Neural Correlates of Semantic Construction During Episodic Memory Recall

**DOI:** 10.1002/hbm.70389

**Published:** 2025-10-25

**Authors:** Carina Zoellner, Rebekka Heinen, Nicole Klein, Nora A. Herweg, Christian J. Merz, Oliver T. Wolf

**Affiliations:** ^1^ Department of Cognitive Psychology, Institute of Cognitive Neuroscience Faculty of Psychology, Ruhr University Bochum Bochum Germany; ^2^ Department of Neuropsychology, Institute of Cognitive Neuroscience, Faculty of Psychology Ruhr University Bochum Bochum Germany

**Keywords:** false memory, fMRI, memory reconstruction, representational similarity, VR

## Abstract

When recalling what you ate for breakfast last Wednesday, you might not remember the exact meal, but you may confidently select the items you typically eat. Here, semantic knowledge (i.e., what you usually eat) contributes to the reconstructive process of episodic memory retrieval (i.e., what you actually ate). In the current fMRI study, we used a highly realistic virtual environment to test this influence of semantic knowledge on episodic memory retrieval. During the task, 60 participants actively (task‐relevant) or passively (task‐irrelevant) encountered everyday objects that were either congruent (i.e., rubber duck in the bathroom) or incongruent (i.e., a toaster in the bathroom) with their expected location. Thereby, we created conflicting information between the episodic memory trace (toaster in the bathroom) and semantic information (toaster in the kitchen) during retrieval. Using multivariate analyses, we analyzed the neural basis of this semantic bias. Further, we administered cortisol, typically associated with impaired episodic memory retrieval, to half of the participants prior to retrieval, thereby manipulating the balance between correct episodic and incorrect semantic retrieval. In the lateral occipital cortex (LOC), incongruent task‐relevant objects showed greater similarity to their congruent semantic counterparts than did task‐irrelevant objects. Notably, spatial memory tended to be reflected in similarity patterns in the LOC. Strikingly, incongruent objects showed a higher pattern reorganization (i.e., pre‐/post‐encoding similarity) compared to congruent objects, reflecting a difference in neural representation for objects encountered in conflict with prior knowledge. In contrast to our hypotheses, cortisol prior to retrieval had no effect on semantic bias. However, cortisol influenced neural pattern similarity: we found higher pattern reorganization within the posterior hippocampus in the cortisol group. Similarly, we found higher confidence to be linked with similarity patterns in the LOC and lingual gyrus in the placebo, but not in the cortisol group. This indicates an effect of cortisol on memory trace reinstatement during retrieval. Our findings on incongruent object processing contribute to the understanding of how the human brain constructs past episodes from episodic memory traces, suggesting an influence of prior semantic knowledge, reflected in neural similarity patterns.

## Graphical abstract

The study used fMRI and a virtual environment to explore how semantic knowledge affects episodic memory retrieval. Results are threefold: the semantic representation of objects increased with better memory, spatial memory was mapped in the lateral occipital cortex, and incongruent objects showed a higher encoding‐retrieval similarity compared to congruent objects.

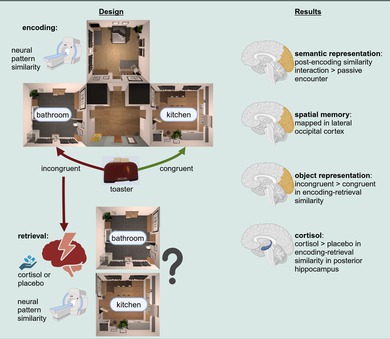

## 1 Introduction

Retrieving past experiences is a crucial aspect of everyday life, as our memories guide actions, inform thoughts, and shape our understanding of the world. However, memories are not merely the reproduction of past episodes but are subject to consistent changes, revealing a level of untrustworthiness. Although humans rely on whatever happened to them during the past day, month, or year, influential behavioral studies have unraveled systematic memory errors and biases during the retrieval process, shaped by various factors such as semantic knowledge (Bartlett 1932; Carpenter and Schacter 2017; Roediger and McDermott 1995). One framework aiming to explain these retrieval errors is the scenario construction model, which states that memory retrieval is in fact a constructive process, to which various systems contribute (Cheng et al. 2016). Gist information is retrieved from what has been encoded and consolidated from the relevant episode. During retrieval, the gist is complemented by situational and personal factors, all contributing to the ability to travel back in time to the specific episode. Various effects of semantic knowledge on episodic memory retrieval have been demonstrated. These range from the false retrieval of semantically related words in word list recall tasks to the retrieval of an incongruent object's location at a semantically fitting but incorrect place after encoding a realistic virtual environment (Roediger and McDermott 1995; Zöllner et al. 2023). Importantly, the latter finding was observed specifically for objects that were encountered passively. Thus, task relevance appears to shift the balance between semantic knowledge and episodic memory in favor of the actual episodic memory trace. Passively perceived items (i.e., task‐irrelevant) show a weaker episodic memory trace compared to objects that were interacted with (task‐relevant), which in turn showed a stronger episodic memory trace as predicted by the enactment effect (Roberts et al. 2022; Zöllner et al. 2023). This effect might be mediated by attentional mechanisms. While the influence of semantic knowledge on the content of episodic memory retrieval has been demonstrated at the behavioral level, its neural correlates remain unclear. In addition to personal factors such as a priori semantic knowledge and personal relevance influencing what is retrieved episodically, situational factors such as current mood and acute stress may further impact episodic memory retrieval and, relatedly, the semantic bias (Shields et al. 2017; Wolf 2017). Prior studies found cortisol to be associated with impaired episodic memory retrieval and decreased brain activity in the hippocampus during retrieval processes (Oei et al. 2007), but it is unclear whether this results in systematic changes in the semantically biased retrieval. Thus, the current study aims to shed light on the balance between semantic information and episodic memory during retrieval and its neural underpinnings, as well as how both personal factors (e.g., attentional mechanisms) and situational factors (e.g., cortisol) can shift this balance in favor of a semantic bias. To this end, we developed a task that introduces a conflict between the two sources of information.

### 1.1 Semantic Bias in Episodic Memory Retrieval

Prior studies investigating the influence of semantic information on episodic memory retrieval have used congruency manipulation, that is, placing some items at odds with their expected location. The findings revealed that prior knowledge typically leads to enhanced memory for congruent compared to incongruent items (Bein et al. 2015; Laurent et al. 2020; van Kesteren et al. 2012; Zöllner et al. 2023). Nevertheless, the likelihood of correctly retrieving incongruent objects can be selectively enhanced by manipulating attentional mechanisms, that is, manipulating task relevance, increasing saliency, or by placing objects in highly surprising locations (Greve et al. 2019; Quent et al. 2022; van Kesteren et al. 2012), resulting in stronger episodic memory traces and consequently a weaker semantic bias. To start with, incongruently encountered objects take up more attention compared to congruent objects during visual scene perception (Coco et al. 2020). Multiple factors determine whether encoded information is transferred into a strong episodic memory trace. In this study, we are specifically interested in the spatial recall of incongruent objects with weaker episodic memory traces. In a prior behavioral study, we demonstrated that incongruent objects which were not placed in their correct episodic location were more likely to be placed in the semantically fitting room rather than in an unrelated one (Zöllner et al. 2023). Thus, we found a two‐fold semantic bias: first, by showing that prior knowledge enhanced memory for congruently encountered objects, and second, by showing that incorrectly placed incongruent objects were more likely to be sorted to the semantically fitting rather than to the unrelated room. Importantly, by manipulating attention and denoting half of the objects as task‐relevant, we demonstrated that semantic bias was stronger for task‐irrelevant objects, for which a weaker episodic memory trace was expected. We predict that the current study can behaviorally replicate these findings.

### 1.2 Neural Correlates of Episodic and Semantic Memory Representations

Disentangling episodic and semantic information at the neural level remains a key challenge. Multivariate approaches such as representational similarity analysis (RSA) allow us to investigate representations of individual events by estimating the similarity of activation patterns across voxels in a region of interest between two stimuli or events (Chadwick et al. 2010; Kriegeskorte and Kievit 2013; LaRocque et al. 2013; Xue 2018). Although various studies using RSA have demonstrated a pivotal role for the hippocampus in episodic memory retrieval (Liang and Preston 2017; Libby et al. 2019; McKenzie et al. 2014), investigating the link between representational similarity in the hippocampus and behavioral memory performance has revealed mixed results. Some studies suggest relative increases (Deuker et al. 2016; Dimsdale‐Zucker et al. 2018), but others find decreases in pattern similarity (LaRocque et al. 2013; Pidgeon and Morcom 2016; Wing et al. 2020). Increased similarity has been observed for stimuli which share a temporal and spatial context, i.e., pattern integration (Deuker et al. 2016; Libby et al. 2019), implicating a binding or integration of context‐related stimuli. However, other studies note a decrease in hippocampal pattern similarity between objects sharing a context, indicating a differentiation (i.e., pattern separation) between individual stimuli to reduce interference during retrieval (Chanales et al. 2017; Dimsdale‐Zucker et al. 2018). Recent findings suggest that in particular, a high overlap in content or context leads to a decrease in similarity for episodically related stimuli (Brunec et al. 2020). These diverse results, regarding both higher and lower representational similarities, can be linked to different processes in hippocampal substructures. The functioning of the hippocampus differs along its long‐axis, with the anterior part being more involved in pattern integration and similarity, and conversely, the posterior part being more involved in separation processes and differentiation. These distinct representational patterns in hippocampal subfields can also be present at the same time (Dimsdale‐Zucker et al. 2018). Additionally, differences in task demand explain hippocampal involvement in both the integration of related memories and pattern separation processes, as both processes can be beneficial for later memory retrieval (Brunec et al. 2020). RSA can further be used to investigate reinstatement effects (i.e., pre to post encoding similarity) by comparing neural pattern similarity across the first encoding of a stimulus and its retrieval, and these effects have been linked to memory accuracy (Bird et al. 2015; Fandakova et al. 2019; Liang and Preston 2017; Tompary and Davachi 2017; Tompary et al. 2016). Thus, neural reactivations of the same stimulus could strengthen differentiated stimulus‐specific representations in the hippocampus. Naturally, memory representations—neural substrates of past events—depend on a wide network of brain regions, among which prefrontal and sensory regions play an important role (Renoult et al. 2019; Tompary and Davachi 2017).

While the role of the hippocampus in episodic memory is widely studied, neural correlates of semantic bias remain sparse. Neural correlates of semantic information are thought to be hippocampus‐independent in most traditional models of memory (Nadel and Moscovitch 1997). However, findings of so‐called concept‐cells, which fire with the perception of a specific semantically consolidated stimulus in the medial temporal lobe, suggest at least a partial involvement of hippocampal structures in the representation of semantic information (Quiroga 2012). Furthermore, there is evidence for hippocampal recruitment in semantic processing tasks when spatial information is required (Sheldon and Moscovitch 2012). The question remains: will representational similarities in the hippocampus reveal differences between episodic and semantically biased event retrieval?

In investigating semantic processing, a special role is attributed to the ventral visual stream (VVS), which has been shown to process object categories (Coggan et al. 2016; Heinen et al. 2024; Kravitz et al. 2013). However, it has been argued that selectivity for semanticized object categories in VVS could also be reduced to shared visual properties instead of semantic relations between objects belonging to the same category (Rice et al. 2014; Watson et al. 2014). Contrastingly, when comparing objects sharing shape or semantic category, higher similarities were observed between objects belonging to the same semantic category compared to objects belonging to different semantic categories in the lateral occipitotemporal cortex (LOC), lingual gyrus, and other parts of VVS, irrespective of their visual similarity (Bartha et al. 2003; op de Beeck et al. 2019; Bracci and op de Beeck 2016; Devereux et al. 2013; Peelen and Downing 2017). These findings cast doubt on the theory that visual feature processing is solely responsible for category‐similarity in VVS. Therefore, whether semantic similarity along the VVS will lead to more errors if a priori knowledge is used during retrieval, instead of episodic memory, remains to be investigated.

### 1.3 Episodic Memory and Stress (Hormones)

In addition to personal factors, situational factors can influence the formation of episodic memory traces, as well as access to them. Stress is a major factor impacting memory. Episodic memory retrieval has been shown to be impaired after exposure to a real or anticipated threat (Shields et al. 2017; Wolf 2017). Studies targeting the diverse biological mechanisms of this effect found that the release of glucocorticoids in particular is associated with impairments in episodic memory retrieval. Inducing real stress in laboratory settings remains challenging, and the pharmacological application of cortisol has been shown to mimic the impairing effects of stress on retrieval in several studies (De Quervain et al. 2000; Kirschbaum et al. 1996; Tollenaar et al. 2009). Impairing effects were, for example, found during tasks that involved free recall (De Quervain et al. 2000; Kuhlmann et al. 2005; Tops et al. 2004), recognition memory (Diekelmann et al. 2011), and spatial memory (Kirschbaum et al. 1996). However, there remains some controversy regarding the generalizability of this effect. Previous findings demonstrate that cortisol intake led to impaired associative memory, specifically in men, but did not affect item memory itself (Antypa et al. 2022; Merz and Wolf 2017). Nongenomic (i.e., rapid) stress hormone effects are associated with an increase in amygdala activity, disruption of prefrontal activity, and enhancement of hippocampal plasticity, leading to an impairment in memory retrieval of unrelated information (Gagnon and Wagner 2016). Contrastingly, genomic (i.e., slow) glucocorticoid effects led to reduced hippocampal plasticity, modulated amygdala and prefrontal functioning, and were associated with impaired memory retrieval of unrelated information as well (Gagnon and Wagner 2016). With regards to semantic bias, Diekelmann et al. (2011) showed not only hindered recognition of correct memories but also a reduction in susceptibility to false memories, arguing that episodic‐based reconstruction of past events might be hindered by pharmacological cortisol administration. Thus, we aimed to investigate whether cortisol, in contrast to task relevance, would increase the semantic bias and shift the balance from episodic retrieval toward an increase in false, semantically biased memories.

### 1.4 Summary

Combining analyses of behavioral and neural response patterns with cortisol administration, the current study investigates memory retrieval when there is conflict between episodic memory and semantic information. To this end, participants navigated through a virtual environment, in which some objects were placed according to their expected semantic category (i.e., a teddy bear in the bedroom; congruent) while others were placed in unusual places (i.e., a toaster in the bathroom; incongruent). We hypothesize that cortisol administration prior to retrieval will impair episodic memory retrieval. We also predict that this impairment will consequently influence the extent to which participants rely on their semantic knowledge and succumb to a semantic bias. This should be reflected in participants placing erroneously retrieved incongruent objects into their semantic (expected) room (spatial memory). We also expect memory performance to be reflected in the neural pattern similarity between objects pre‐ compared to post‐encoding (pattern reorganization), estimated with functional magnetic resonance imaging (fMRI). We expect pattern similarity to be shaped by semantic category affiliation in regions relevant for semantic memory representation pre‐encoding. Post‐encoding, we hypothesize that pattern similarity will be predicted by the participants' experience during encoding. However, since cortisol is expected to hinder memory retrieval and not impact the consolidated memory representation itself, we predict that the accuracy of behavioral memory retrieval will be a better predictor of neural pattern similarity in the placebo group but a worse predictor of memory performance in the cortisol group. To investigate neural correlates of episodic memory, we focused on the anterior and the posterior hippocampus (aHC and pHC). For the semantic bias, we targeted the LOC and the lingual gyrus as markers for semantic object category selectivity. Considering that memory retrieval is widely distributed across the brain, we conducted a whole‐brain searchlight analysis to further understand which regions are involved when individuals rely on prior semantic knowledge during uncertain situations.

## 2 Materials and Methods

The study was approved by the local medical ethics committee of the Ruhr University Bochum, Reg.‐No. 18‐6368 and was conducted in accordance with the Declaration of Helsinki. The current study and analysis were preregistered in a project at the Open Science Framework (OSF), accessible under the link: https://osf.io/zvr2n/?view_only=9992edc23e7b45bd99bae9bdeecf6f3c. We share raw data and code under the following link: https://osf.io/5k3mx/.

### 2.1 Sample

Our sample consisted of *N* = 60 healthy, male participants, all of whom fulfilled predefined inclusion criteria: We only included right‐handed participants, aged between 18 and 35 years with a normal BMI between 18 and 30 kg/m^2^. We ensured fluency in the German language for the sake of our language‐dependent tasks. All participants were in good general health (i.e., no acute or chronic, currently treated or impairing diseases, and no history of psychiatric or neurological diseases) and had normal or corrected‐to‐normal vision. We excluded participants suffering from motion sickness to avoid distractions during the virtual game task. Since we manipulated cortisol levels through cortisol intake and since we wanted to exclude further influences from varying hormone levels between participants: participants were male (i.e., not influenced by the female hormonal cycle, Merz and Wolf 2017), reported no regular hormone intake and did not work in night shifts. Low‐risk fMRI measurements were ensured by participants being free of non‐removable metal, hearing impairments, fear of small spaces, prior occupation in a metal‐working environment, and tattoos not classified as risk‐free.

We estimated the sample size by conducting an a priori power analysis using G*Power (Faul et al. 2007) with a medium effect of *f* = 0.3, a Type I error of *α* = 0.0001 (acknowledging that correction for multiple comparisons was probably necessary for fMRI analyses) and a power of 1 − β = 0.8 for a repeated‐measures ANOVA with a within‐between interaction for two groups (cortisol/placebo) and 4 repeated measures (congruent/incongruent × task‐relevant/task‐irrelevant). The resulting sample size of 58 was increased to 60 for reasons of expected drop‐out. Reasons for exclusion during ongoing data acquisition on the first day were technical failure (*N* = 1) and sickness or indisposition of participants (*N* = 4). Excluded participants (*N* = 5) were replaced with additionally collected participants. Thus, we have a final sample of 60 participants. Especially in light of a lack of reliable functions to calculate fMRI power for RSA, we made sure that this number was consistent with previous studies using the same behavioral design (Zöllner et al. 2023) or analysis methods, specifically RSA (Deuker et al. 2016; Lim et al. 2023) and cortisol application (Het et al. 2005). Participants were right‐handed and aged between 18 and 34 years (*M* = 24.38, SD = 4.10). Importantly, both groups were comparable with regard to age (cortisol: *M* = 24.53, SD = 4.23, placebo: *M* = 24.23, SD = 4.03; *t*(57.864) = 0.281, *p* = 0.780, *d* = 0.073), body mass index (BMI, all: *M* = 24.16, SD = 2.72, cortisol: *M* = 24.00, SD = 2.62, placebo: *M* = 24.31, SD = 2.85; *t*(57.579) = 0.432, *p* = 0.668, *d* = 0.111) and gaming experience (all: 55% with‐, 45% without regular experience, cortisol: 56.67% / 43.33%, placebo: 53.33%/46.67%; Χ2(1) = 0, *p* = 1, w = 0.03). Regular gaming experience comprised gaming on a 4‐level scale from at least once per month up to several hours a day (monthly, weekly, several times per week, daily). To ensure that the level of gaming experience did not influence subsequent memory, we conducted ANOVAs predicting memory performance, which revealed no effect of gaming experience on later memory (included in the Supporting Information S2).

All participants received compensation of 10€ per hour or course credits. We advertised our study at the Ruhr University Bochum, using social media channels and other local resources.

### 2.2 Cortisol Administration

Participants were randomly assigned to either the placebo (*N* = 30) or the cortisol group (*N* = 30). Each participant in the cortisol group received two 10 mg hydrocortisone tablets on the second day of the experiment, while the placebo group received two tablets of a visually identical placebo. Experimenters were not aware of the participants' group affiliation (double‐blind). To assess physiological responses following the intake of cortisol or placebo, we collected saliva samples using Salivettes (Sarstedt, Nümbrecht, Germany) at the following points in time with respect to tablet intake: *T* − 2 (baseline), *T* + 30, *T* + 95, *T* + 105. Saliva samples were stored at −20°C until analyzed with a commercial enzyme‐linked immunosorbent assay (IBL International, Hamburg, Germany). Intra‐assay coefficients of variation (CVs) were below 6.5% and inter‐assay CVs below 8.25%. To control for everyday influences on cortisol levels, we asked participants to refrain from doing unusually intensive sports, consuming alcohol, drugs, or painkillers 1 day prior to and on testing days. Food intake should have taken place prior to measurement, but on the day of cortisol administration not within 1.5 h before. All measurements were realized between 1 p.m. and 8 p.m. to control for effects of the circadian rhythm on cortisol levels.

### 2.3 Materials and Virtual Environment

**FIGURE 1:**
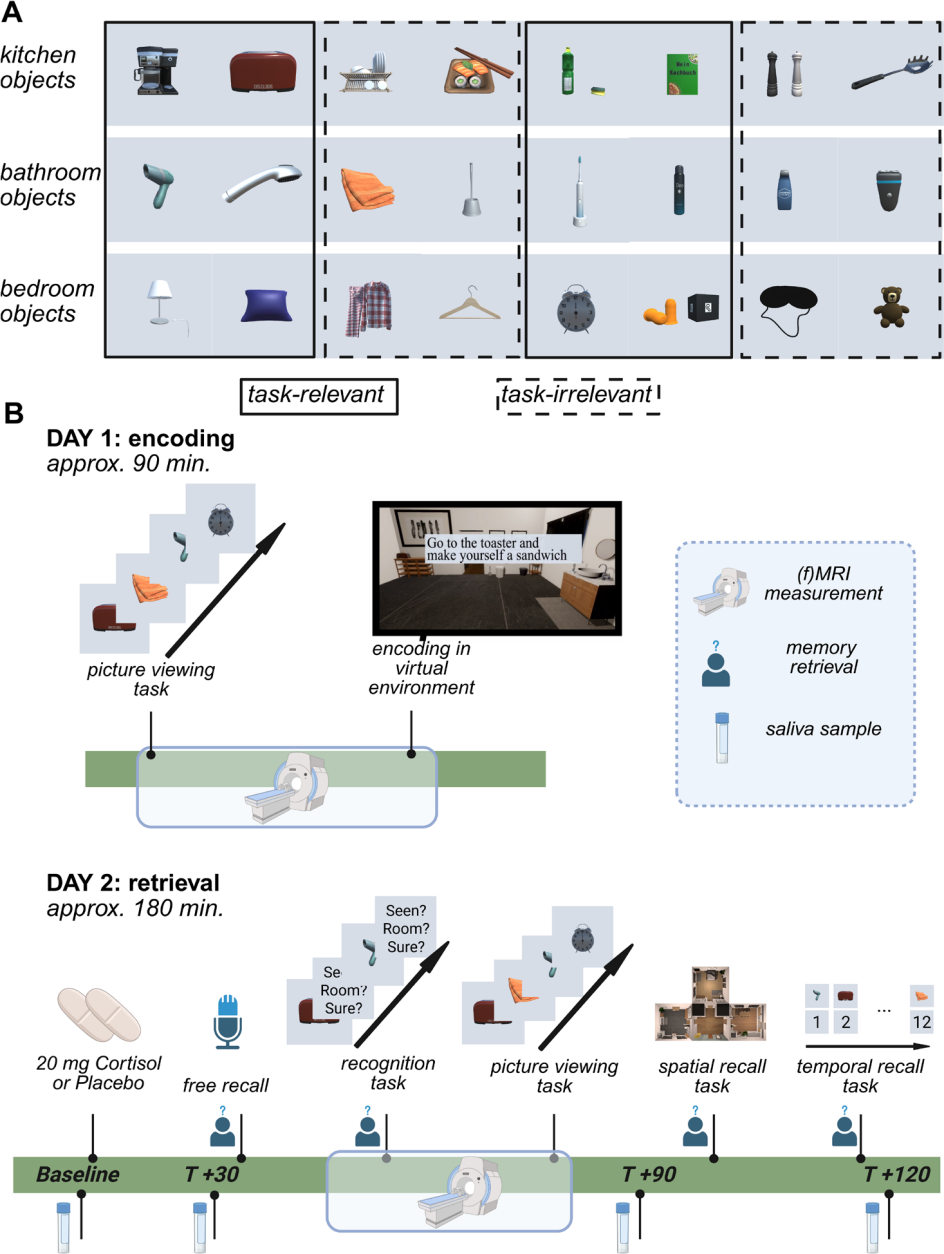
Overview of experimental procedure. (A) Overview of objects central to encoding and retrieval, sorted by semantic room affiliation. All objects were included in the virtual environment during retrieval. We randomly had objects appear congruent or incongruent. Half of the objects were task‐relevant (solid line), the other half was task‐irrelevant (dashed line). (B) Visualized is the methodology from the first two of three days of data collection. Data collection on the first day (PVT‐pre‐encoding) took place in the MR scanner. The second day of data collection (retrieval) was only partly conducted in the MR scanner (recognition task and PVT‐post‐encoding). Prior to fMRI measurements, participants received cortisol or placebo treatment, had a waiting period of 30 min, and conducted a free recall. After fMRI measurements, participants underwent the spatial recall task (SRT) and temporal recall task. Not visualized but analog to the first retrieval is the second retrieval, which was conducted online 28 days after encoding.

The encoding took place in a virtual environment (encoding virtual environment task, EVE task). The use of a virtual environment combines the advantages of having high experimental control applicable in fMRI settings but also preserving ecological validity (S. A. Smith 2019). Using the game engine Unity (Version 2019.1.3, Unity Technologies, San Francisco), we designed a virtual three‐room apartment, consisting of a characteristically furnished kitchen, bathroom, and bedroom, in addition to a neutrally furnished corridor. Packages acquired for this purpose are cited in the Supporting Information S3. During encoding, participants navigated through the environment using an MR‐compatible button box and conducted a series of 12 tasks within the environment (i.e., ‘Go to the toaster and make yourself a sandwich’). In total, 24 objects were placed in the three rooms of the virtual apartment. The objects were either part of acquired packages from the Unity Asset Store or self‐made in the 3D‐modeling program Blender (Version 2.83, https://www.blender.org). An overview of objects is provided in Figure 1A.

### 2.4 Encoding Task

During encoding, participants navigated through a virtual apartment using an MR compatible button box and saw the screen using a mirror system. In the virtual apartment, participants sequentially conducted 12 tasks. Each of the tasks focused on a specific object that was placed in the environment. Participants were first asked to search for the object, approach it, and press a button for 5 s in order to complete the task. A progress bar with an accompanying note visualized the duration of each task. The tasks were meaningful, that is, ran under the cover story of cleaning up the apartment for a date (e.g., “Go to the toaster and make yourself a sandwich.”, see Supporting Information S1 for an overview of all tasks). Half of the objects included in the virtual apartment were relevant to the sequence of actions (task‐relevant) and half of them were just encountered passively (task‐irrelevant). Importantly, the version used in this study had fixed task‐relevant objects and fixed task‐irrelevant objects (i.e., across participants, the coffee machine was always task‐relevant while the sleeping mask was task‐irrelevant. This is depicted with dashed and solid lines in Figure 1A). The environment and stimuli have been previously used in Zöllner et al. (2023). In a behavioral replication study, we verified that random task relevance assignments among objects resulted in the same behavioral pattern (Zöllner et al. 2023). Additionally, we validated category affiliation of objects in a separate pilot study, included in Supporting Information S1. That is, objects appeared either in a congruent/expected or in an incongruent/other‐than‐expected room. For each participant, the objects were pseudo‐randomly placed across the three rooms, with four congruent and four incongruent, and likewise four task‐relevant and four task‐irrelevant objects per room, equally divided among congruent and incongruent object placements and balanced for semantic room affiliation. Prior to the main encoding task, participants underwent a tryout phase of approximately 6 min, in which they were placed in the same apartment, learned how to navigate in it, and encountered four example objects, two of which they interacted with. Importantly, the objects from the tryout phase were later used as example objects to illustrate how the spatial recall task works.

### 2.5 Experimental Procedure

Data collection took place between 08/2021 and 10/2022. The experimental procedure included 3 days of data collection, two of which were consecutive and in‐person at the University Hospital Bergmannsheil in Bochum. The last session took place 28 days after the first session and was conducted online. An overview of the experimental procedure is provided in Figure 1B. Note that we report more detailed methods and respective analyses for the free recall task, temporal recall task, and late recall in the Supporting Information S1 and S2, as they are not central to the claims of this paper and served as supporting analyses.

*Day 1*: Upon arrival on the first day of data acquisition, participants gave written informed consent and were prepared for the fMRI measurement. During fMRI data acquisition, two blocks of a picture viewing task (PVT‐pre) were conducted. In short, PVT consisted of two scanning sessions, each of which contained six blocks during which images were presented. Each block contained 24 pictures of objects. Thus, each of the 24 objects was presented 12 times during PVT. The depicted objects were central to later encoding, as they were part of the encoded episode. Participants had the task to search each picture for a fly and were asked to be furthermore attentive to the objects. Flies were included in 5% of images, which were later excluded from further analyses.

The EVE task followed.

Participants took, on average, *M* = 8.271 min (SD = 1.751) to conduct all tasks (with *M* = 37.815 s, SD = 31.432, per task). Prior to the EVE task, participants had the chance to get to know the controls and environment. To this end, we placed four (purely congruent) example objects in the environment, two of which were task‐relevant, which gave participants a chance to get accustomed to the controls. This exploration phase lasted approximately 6 min and took place during structural T1‐weighted image acquisition.

*Day 2*: The second day included cortisol or placebo administration and saliva sample collection at four different timepoints. Each saliva sample was accompanied by filling in the Positive and Negative Affect Scale (PANAS, Breyer and Bluemke 2016). Prior to administration, participants provided a baseline saliva sample. A participant then had a 30 min waiting period for cortisol concentrations to rise. During that time, a short demographic questionnaire was presented, participants generated an individual code in order to match their online responses reliably, they read the instructions for the subsequent task and were prepared for the fMRI measurement. In the remaining time participants either solved a sudoku puzzle or colored a mandala, depending on their free choice (47 participants chose a sudoku). Afterwards, participants provided a second saliva sample.

Next, participants were brought to the MRI scanner and confronted with a standard recognition task during the first fMRI session. During this task, pictures of the 24 encoded objects and respectively 24 realistic lures were presented (see Supporting Information S1). Each image presentation was followed by an old‐new rating, questioning whether the object was part of the episode or not. In cases of old ratings, participants indicated which room they remembered to have encountered the object. Finally, we asked participants for their confidence in room sorting. After recognition, participants were again confronted with two fMRI sessions of PVT (PVT‐post). Outside of the scanner, participants provided a third saliva sample and were then asked to first conduct a spatial recall task (SRT). The SRT was a drag‐and‐drop task in which participants were asked to sort all 24 objects to the location they remembered to have seen them on a birds‐eye view depiction of the virtual apartment they encoded on the day before. They were explicitly instructed that, despite the change in perspective, the depiction of the apartment and furniture is exactly equal to the apartment they experienced the day before. Participants were free to place the objects in whatever order they preferred. A final saliva sample concluded the testing day. Behavioral pre‐ and post‐fMRI procedures took place in a preparation room at the hospital.

### 2.6 Preprocessing and Statistical Analyses

*Software*: We used Python (3.0.0) to prepare raw data for further analyses and used pandas (McKinney 2010) and numpy (Harris et al. 2020). (f)MRI data were prepared and preprocessed with MATLAB (2020b, https://www.mathworks.com/) based SPM12 (FIL Methods Group) toolbox. We used FreeSurfer (v6.0.0, https://surfer.nmr.mgh.harvard.edu/) for brain extraction. Searchlight analyses were further conducted with FSL (Jenkinson et al. 2012). We used Python‐implemented scikit‐learn toolbox (Pedregosa 2011) for multidimensional scaling and plotted results with matplotlib (Hunter 2007). All statistical analyses and graphical data visualization were conducted using R (R Core Team 2022) implementation in R‐Studio (RStudio Team 2020). All R‐packages in use for our analyses are summarized in the Supporting Information S4. Figures and parts of the stimulus material were designed with inkscape (Inkscape 2022) and biorender (http://biorender.com).

*Statistical approaches*: To compare mean values between or within subjects, we conducted *t*‐test comparisons reporting effect sizes calculated by Cohen's *d* or conducted Wilcoxon Signed Rank tests using R‐package rstatix (Kassambara 2021) as a non‐parametric alternative when assumptions for *t*‐tests were violated. For multi‐factor comparisons of mean values, we used ANOVAs using R‐package afex (Singmann et al. 2016), reporting effect sizes with partial eta square (pes). We estimated linear mixed models (LMMs) for analyses at the object level. Within each LMM, we accounted for clustering within individual participants by setting subjects as higher‐level terms, consequently allowing for random intercepts and controlling for this random factor. We used the lmer‐function from the R‐package lmerTest (Kuznetsova et al. 2017) to fit the model and used lmer‐Test anova‐function to estimate the significance of individual model parameters. Thus, *F*‐values were estimated by Satterthwaite's degrees of freedom method. We used type III sum of squares to estimate effects while controlling for other main effects or interaction parameters. We made sure that the variance inflation factor did not exceed 5 to avoid multicollinearity. For LMMs, we calculate Cohen's *f* ^2^ as effect sizes using the R‐package effectsize (Ben‐Shachar et al. 2020). Cohen's *f* ^2^ indicates the proportion of variance an individual predictor accounts for and has been specifically suggested for the use of LMMs (Lorah 2018). Across models, we applied the 0.05 significance threshold to assess significance and used Holm correction to correct for multiple comparisons. Additionally, we included the predictor ‘group‐affiliation’ in all models concerning data acquired on day 2 to analyze effects of cortisol.

*Behavioral Data Analysis*: We sought to replicate the behavioral findings from our previous study (Zöllner et al. 2023). We excluded objects which were not recognized as ‘old’ in the recognition task from all further behavioral analyses. On average, participants retrieved 18.41 of 24 objects (min = 10, max = 23), which resulted in a total number of 1105 objects being the basis of this analysis (thus, on average 5.58 (min = 1, max = 14) and a total of 335 objects were excluded as they were not recognized). We first analyzed whether congruence (a predictor with two levels, for example, the coffee machine in the kitchen—congruent, vs. the toaster in the bathroom—incongruent) and task relevance (likewise a predictor with two levels, for example, the toaster, which was part of the sequence of action—task‐relevant, vs. the towel, which was passively encountered but not interacted with—task‐irrelevant) could predict memory accuracy in the SRT. To this end, we estimated an LMM with congruence and task relevance predicting drop error (i.e., distance between the correct and dropped location). Then, we looked at room sortings and analyzed whether incongruent objects were proportionally more likely to be placed in the semantically fitting room rather than in the unrelated room, separately for task‐relevant and task‐irrelevant objects. With regards to the exclusion of incongruent objects based on the recognition task, participants did not recognize 2.596 incongruent objects (min = 1, max = 7), leaving 9.533 objects as the basis for this analysis (min = 5, max = 12). As the proportions of room placements in spatial recall were not normally distributed, we used Wilcoxon Signed Rank tests for statistical comparisons.

### 2.7 Physiological Data Analysis

First, we checked whether cortisol concentrations increased in the cortisol group compared to the placebo group. Following standard procedures, we aimed at excluding data deviating more than three standard deviations and checked for normality. There was no data excluded based on this criterion. However, there was a drop‐out of *N* = 12 data points from *N* = 10 participants in consequence of non‐analyzability of the saliva samples (i.e., two participants had only *N* = 2 analyzable samples). Thus, the resulting data set consisted of 58 samples at baseline (cortisol: 28 samples, placebo: 30 samples), 56 samples at +30 min. (cortisol: 26 samples, placebo: 30 samples), 59 samples at +90 min. (cortisol: 30 samples, placebo: 29 samples), and 55 samples at +120 min. (cortisol: 2 samples, placebo: 30 samples). Thus, no participant had to be fully excluded based on overall physiological data analysis. Subsequently, an LMM controlling for the random factor subject was conducted with cortisol concentrations predicted by time, group affiliation, and an interaction of both. For post hoc comparisons, we conducted pairwise *t*‐tests.

### 2.8 fMRI

*Acquisition*: MRI images were acquired using a 3T scanner with a 32‐channel head coil (Philips Achieva 3.0 T X‐Series, Philips, the Netherlands). Blood oxygenation level‐dependent (BOLD) contrast images were acquired with a T2‐weighted gradient echo EPI sequence with 2.5 mm isotropic resolution (TR = 2500 ms, TE = 30 ms, FA = 90°, FOV = 96 mm × 96 mm, 45 transversal slices in ascending order without slice gap). The acquisition time (TA) differed between the tasks. Additionally, a high‐resolution whole‐brain structural brain scan was acquired using a T1‐weighted sequence at 1 mm isotropic resolution (FOV: 240 mm × 240 mm, transversally oriented slices) with a TA of 6 min 2 s. In total, participants underwent six functional runs—two during an initial picture viewing task (PVT‐pre), one during the encoding phase (EVE task), one at the second day during the recognition task, and finally two during another PVT(‐post).

*ROI selection*: Subject‐specific anatomical regions of interest (ROIs) were extracted from structural scans using the parcellation procedure embedded in FreeSurfer (v6.0.0, https://surfer.nmr.mgh.harvard.edu/). Extracted ROIs were based on the Desikan–Killiany atlas (Desikan et al. 2006). Specifically, we chose the hippocampus (and separated it into anterior and posterior), lingual gyrus, LOC, and ventral visual stream (parahippocampal, pericalcarine, cuneus, fusiform gyrus, lingual gyrus, and LOC).

**FIGURE 2:**
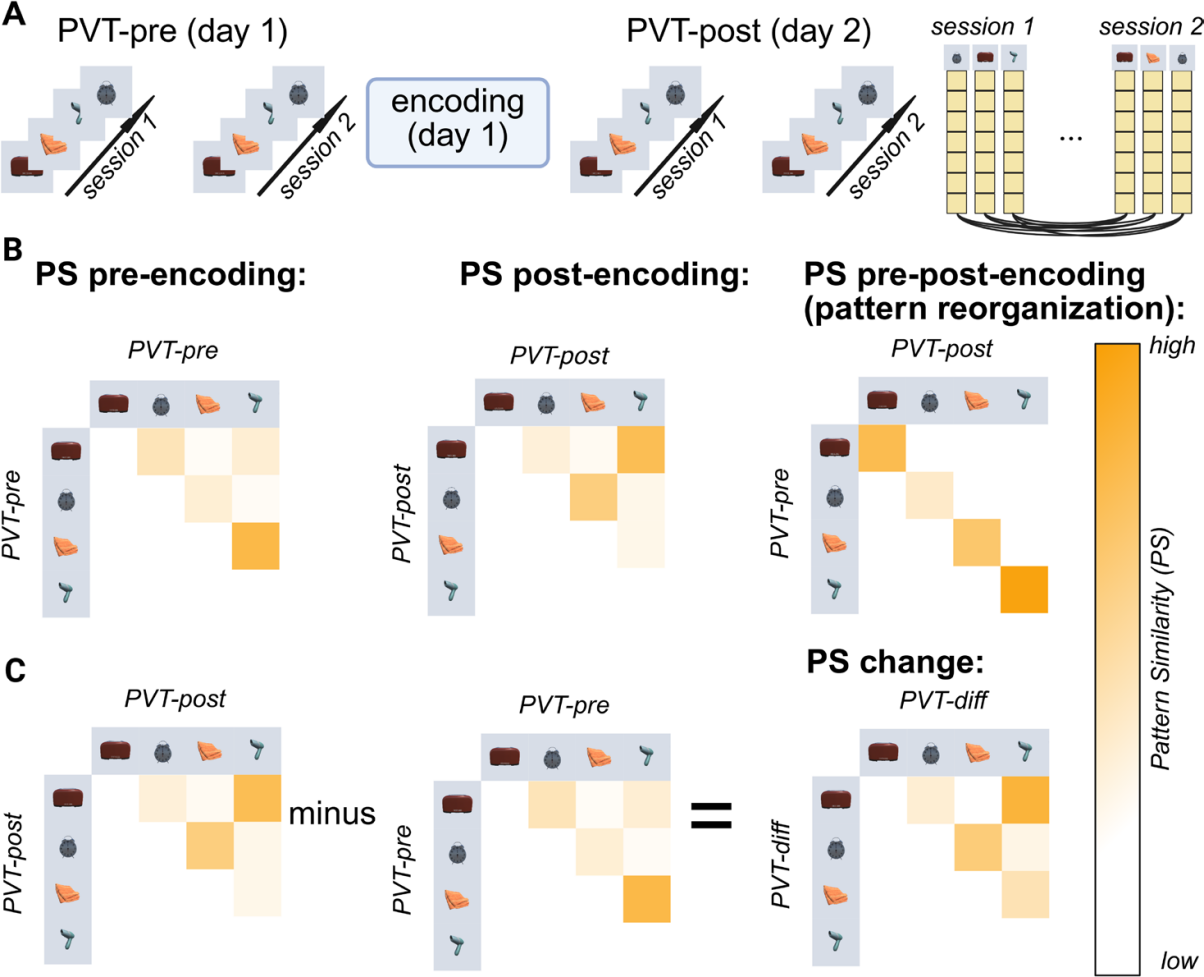
Visualization of fMRI analysis approaches to obtain neural similarity patterns. Depicted is a visualization for fMRI pattern similarity (PS) values included in analyses. (A) The basis of our PS analyses are all four PVT sessions, two were conducted pre‐ and two post‐encoding. For each ROI and searchlight sphere, we extracted all voxel‐activations for each object's presentation. Voxel activations for each object were averaged within sessions for repeated presentations. We estimated Spearman's R for each object's voxel activation pattern between sessions within 1 day or between days. (B) We analyzed PS between object pairs pre‐encoding (PS pre‐encoding), PS post‐encoding and PS for object pairs pre‐ correlated with post‐encoding (PS pre‐post‐encoding, i.e., pattern reorganization). (C) We estimated representational change using the difference value between PS post‐ and PS pre‐encoding (PS change).

*Multivariate Pattern Analysis within ROIs*: The current study focuses on the fMRI data from the PVT to analyze the (change in) neural object representations after encoding and relate it to behavioral recall data (see Figure 2A). We preprocessed the data using SPM12 (FIL Methods Group). Preprocessing included slice time correction and spatial realignment, but no spatial smoothing in order to conduct multivariate pattern analyses (MVPA). We subsequently coregistered all ROIs to functional space. For MVPA, we have set up a general linear model modeling all objects, the fly, and motion parameters as regressors. To account for motion, we estimated D‐Vars and framewise displacement (FD) values for each volume for each participant. We applied the approach from Afyouni and Nichols (2018) and estimated significantly affected volumes. Those were inserted as additional regressors in our GLM, eliminating influences due to excessive motion. Participants, for whom in consequence less than two presentations of an object remained, were excluded from further analyses. Additionally, participants who missed more than three flies in one of the four runs were excluded from further analyses. This resulted in a total exclusion of: PVT‐pre: *N* = 3, PVT‐post: *N = 6*, PVT‐change: *N* = 9. To additionally reduce the influence of noisy voxels, we obtained t‐values by dividing resulting beta‐maps with the residual matrix.

Across all voxels of a ROI, we correlated each object's activation estimate of one run with each object's estimates from the other run for each day separately, estimating Spearman's correlation coefficient. We used Fisher z‐standardization on the correlation patterns (see Figure 2A). For each ROI, we thereby obtained one correlation matrix across objects for each day (i.e., day 1 (pre‐encoding): object × object and day 2 (post‐encoding): object × object), reflecting pattern reorganization (pre‐post‐encoding similarity: see Figure 2B). To analyze representational change (i.e., what has changed between pre‐encoding PVT and post‐encoding PVT), we subtracted the pre‐encoding matrix from the post‐encoding matrix to obtain a PS‐change matrix (see Figure 2C). In an additional exploratory approach, we analyzed how similar an object was represented to itself pre‐ compared to post‐encoding. For this, we co‐registered post‐encoding betas to pre‐encoding functional space and calculated representational similarity with the upstanding procedure between pre‐ and post‐encoding object presentation. Importantly, we correlated activation estimates of both sessions on the first day with each session on the second day and estimated the average. Thus, we obtained a cross‐correlation matrix representing how similar an object was to itself pre‐ versus post‐encoding (see Figure 2B).

*Statistical analyses of fMRI data*: First, we checked whether pattern similarity pre‐encoding (day 1) could be predicted by semantic category affiliation, specifically in the VVS overall, in the LOC and the lingual gyrus as relevant parts of the VVS for semantic representation, and the hippocampus (HC). To this end, we used an LMM approach including subjects as random factors. This analysis was conducted on *N* = 57 participants, with a total of 15,732 object pairs (276 object pairs per participant; all possible object combinations of 24 objects without duplicates). For each ROI, representational similarity scores for all object pairs were predicted by semantic room category with the two levels ‘same semantic room category’ and ‘different semantic room categories’. We additionally conducted multidimensional scaling as a visualization approach (Lin et al. 2019). That is, the higher dimensional space (i.e., pairwise similarity matrices for all participants) was mapped to a lower‐dimensional space (i.e., two‐dimensional space using Euclidean distance), while the distance between points was preserved as much as possible.

Next, we looked at pattern similarity post‐encoding. In light of the key manipulation in this study design (i.e., presenting objects incongruently and investigating whether objects were retrieved correctly or according to their semantic category), we specifically looked at the neural pattern similarity of an incongruent object to congruent objects of the same semantic category post encoding (i.e., how similar was the toothbrush presented in the kitchen to the hairbrush, shampoo, towel, and hairdryer presented in the bathroom?). How did the semantic representation of an object change following the incongruent encounter? We used two approaches to compare neural pattern similarity at retrieval in ROIs relevant for memory representation (i.e., pHC and aHC, lingual gyrus, and LOC): First, according to our behavioral findings in the previous study (Zöllner et al. 2023), task‐relevant objects showed increased memory performance. Thus, we compared mean pattern similarity of incongruent task‐relevant objects to their semantically congruent counterparts with mean pattern similarity of incongruent task‐irrelevant objects to their semantically congruent counterparts. The second approach focuses on the room‐choice of participants. We compared mean pattern similarity of correctly retrieved incongruent objects to their semantic congruent counterparts with mean pattern similarity of incongruent objects placed in the semantically fitting room to their semantic congruent counterparts. In this way, we can estimate how a semantic choice in cases of weak episodic memory traces is represented differently compared to a stronger episodic memory trace. Post‐encoding analyses were based on *N* = 54 participants (*N* = 27 per group). Of note, all participants had six incongruent task‐relevant and six incongruent task‐irrelevant objects. Regarding room‐choices of incongruent objects, participants had an average of *M* = 4.067 sortings to the semantic room (min = 1, max = 9) and *M* = 6.217 sortings to the correct room (min = 3, max = 10). In detail, we calculated the average pairwise pattern similarity of an incongruent object to all congruently encountered objects of the same category (the average value was based on four pairwise similarity values per incongruent object). Subsequently, we calculated average values across estimated means of all incongruent objects separately for each condition (task‐relevant vs. task‐irrelevant and semantic vs. correct). Then, we conducted mixed ANOVAs predicting mean pattern similarity with task relevance (task‐relevant vs. task‐irrelevant), group‐affiliation (placebo vs. cortisol), and an interaction of both predictors (task relevance by group). The same statistical design was used to predict mean pattern similarity with room‐choice (correct vs. semantic), group‐affiliation, and an interaction of both predictors. In this specific analysis, we did not exclude objects that were not recognized in the recognition task because this analysis focused on the semantic representation of incongruent objects.

In the next step, we looked at pairwise pattern similarity change between objects predicted by the respective variable of interest. After excluding all object pairs in which at least one object was not recognized in the recognition task, these analyses were based on a total of 8411 object pairs from *N* = 51 participants (cortisol group: *N* = 26, placebo group: *N* = 25; object pairs per participant: *M* = 164.92; SD = 52.57). Chosen ROIs were the aHC and the pHC as ROIs relevant for episodic memory representation, and the LOC and the lingual gyrus as ROIs relevant for semantic memory representation. As preregistered, we were specifically interested in whether spatial retrieval could predict pairwise pattern similarity change between two objects. To this end, for each ROI, we estimated two LMMs, in each of which cortisol group affiliation was included as a covariate: in a first model, we included the dichotomous predictor ‘same or different room recall’, and in a second model, we included the continuous predictor ‘proximity of objects in spatial recall’, because two objects might have been retrieved in the same room, but on completely opposite corners of this room, while two other objects might have been recalled having been directly next to each other. Exploratorily, we set up a third LMM and looked at whether average confidence of spatial recall (i.e., how sure a participant was with each chosen location) influenced pattern similarity change for object pairs. Going beyond our preregistered analysis, we were furthermore interested in whether the representation of an object changed comparing pre‐ vs. post‐encoding. Again, excluding objects which were not recognized in the recognition task resulted in a total of 939 objects from *N* = 51 participants (*M* = 18.41; SD = 3.07 objects per participant). To this end, we estimated the neural pattern similarity of an object pre‐encoding with itself post‐encoding. We then related pattern similarity to our memory measures, object characteristics, and cortisol group affiliation. Thus, we estimated five models for each ROI. In each LMM, cortisol group affiliation was included as a covariate. In the first model, we predicted pattern reorganization with the dichotomous predictor “correct room recall.” The second model used the continuous predictor “drop error.” The third model predicted pattern reorganization with confidence of placement in SRT. A fourth model was estimated predicting pattern reorganization with the dichotomous predictor “congruence”, and a final model was estimated with the predictor variable “task relevance.”

Of note, full model output tables for each analysis and model specifications are included in Supporting Information S6–S9.

*Multivariate pattern analysis in searchlights*: We conducted a whole‐brain searchlight analysis in addition to ROI analyses. We used previously extracted beta estimates from the first day and co‐registered the beta estimates from the second day to the functional space of the first day. Again, we obtained *t*‐values to control for noisy voxels. For the searchlight analysis, only gray‐matter voxels were included by using the subject‐specific gray matter mask segmented with FreeSurfer (v6.0.0, https://surfer.nmr.mgh.harvard.edu/). Moving through all voxels, we defined a sphere radius of 6 voxels, centered at each respective voxel. A sphere had to consist of at least 20 voxels to be analyzed further. Each valid sphere was then treated as a ROI, and, following the same procedure as in the RSA ROI analyses, we extracted similarity matrices pre‐encoding and for an object to itself pre‐ versus post‐encoding for each searchlight sphere, mimicking the procedure as in previous ROI analyses. We refrained from analyzing representational change in searchlight for reasons of limited interpretability. We estimated statistically whether pre‐encoding similarity between objects could predict category affiliation (i.e., same/different semantic room categories). We analyzed whether the similarity of an object to itself pre‐ compared to post‐encoding could predict memory retrieval (i.e., correct‐/incorrect room) or the characteristic of object encounter (i.e., congruent/incongruent and task‐relevant/task‐irrelevant). Model estimates (i.e., *t*‐values for dichotomous predictors or correlation values for continuous predictors estimated with MATLAB's corr2 function) were saved for each sphere location to perform *t*‐testing against zero. For testing two conditions against each other, we saved the mean activation for dichotomous predictors for both levels within a sphere, to later statistically contrast the two searchlight maps for each dichotomous predictor. Whole‐brain model estimate maps were then normalized to MNI standard space with SPM12 in order to conduct group‐level analyses.

For analyses, a non‐parametric version of the one‐sample t‐test was conducted with *randomize* (Winkler et al. 2014) in FSL (version 5.0.9, http://fsl.fmrib.ox.ac.uk/fsl/fslwiki/) to estimate whether model estimate maps significantly differed from zero in searchlight spheres. In a second step, we conducted a two‐sample paired *t*‐test, specifically contrasting activation patterns of dichotomous predictors in pattern reorganization similarity matrices. Specifically, 5000 random sign‐flips and threshold‐free cluster enhancement (TFCE) were used to identify significant clusters across participants (S. M. Smith and Nichols 2009). TFCE takes both voxel‐wise signal strength and extent of neighboring voxels into account, while not using an absolute threshold. Results were family‐wise error (FEW) corrected and thresholded at *p* < 0.05 to control for multiple comparisons. Anatomical labels were taken from the Harvard‐Oxford Cortical Structural atlas (Desikan et al. 2006).

## 3 Results

### 3.1 Cortisol Concentrations and Negative Affect

We found that while cortisol concentrations increased over time in the cortisol group (all *t* > 7.670, all *p*_*Holm*_ < 0.001), resulting in a significant group difference at all timepoints except at baseline (baseline: *t*(159) = 0.698, *p*_*Holm*_ = 1, other timepoints: all *t* > 12.843, all *p*_*Holm*_ < 0.001), the placebo group showed a decrease in cortisol between baseline and later timepoints (+90 min, *t*(163) = 3.461, *p*_*Holm*_ < 0.01; +120 min, *t*(163) = 3.256, *p*_*Holm*_ < 0.05), reflecting the circadian rhythm. Neither was there a significant group difference in reported negative affect (*F*(1, 57.977) = 2.673, *p* = 0.107, *f* ^2^ = 0.05), which we acquired as a control measure, nor did we find a significant time × group interaction (*F*(3, 163.071) = 1.712, *p* = 0.167, *f* ^2^ = 0.03). Due to the pharmaceutical application of cortisol and as shown in our cortisol manipulation check above, we did not have any cortisol non‐responders. A detailed overview of the statistical analyses regarding cortisol concentrations and negative affect is included in Supporting Information S5.

### 3.2 Replication of Behavioral Study Results—Validity of Design

**FIGURE 3:**
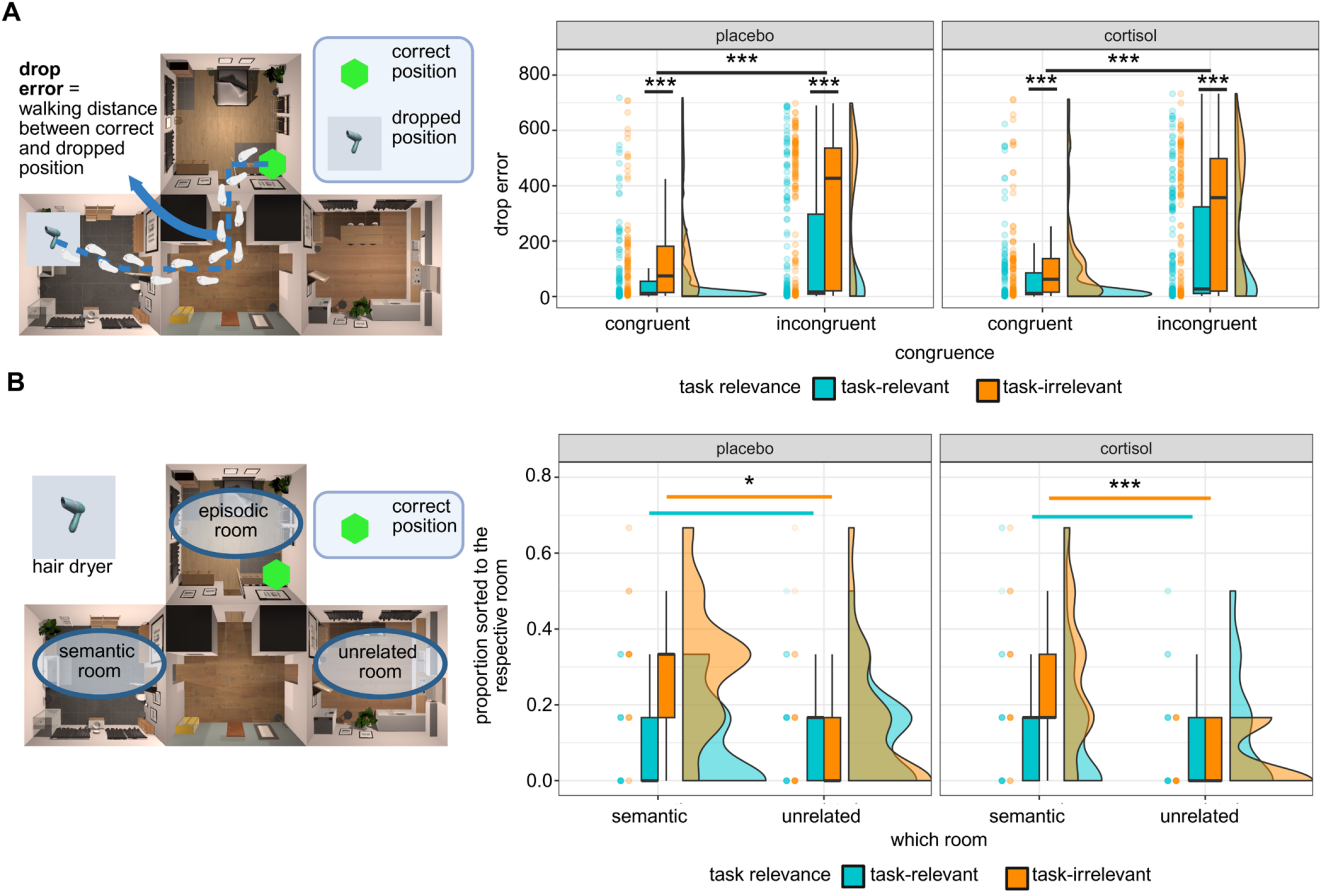
Behavioral results. (A) Distance from the dropped position to the correct position during spatial recall, depending on task relevance and congruence of an object. Task‐relevant object locations were significantly better retrieved than task‐irrelevant objects, and congruent objects were significantly better retrieved than incongruent objects. There was no difference between the cortisol and placebo group. (B) Proportions of room sorting for each participant, separately for task‐relevant and task‐irrelevant objects. The proportion of task‐irrelevant objects sorted to the semantic room was significantly higher than the sorting to the unrelated room. There was no difference between the cortisol and placebo group.*p < 0.05; ***p < 0.001.

Overall, we were able to replicate the findings from our previous study (Zöllner et al. 2023), validating the paradigm. Our previous analyses were expanded by considering group affiliation with regard to cortisol administration. We looked at drop error as a measure for spatial retrieval accuracy and at the semantic bias estimated from the SRT (Figure 3A).

On a behavioral level, drop error was significantly lower for congruent as compared to incongruent objects (*F*_(1, 1380.9)_ = 207.013, *p* < 0.001, *f* ^2^ = 0.15) and for task‐relevant as compared to task‐irrelevant objects (*F*_(1, 1428.5)_ = 41.157, *p* < 0.001, *f* ^2^ = 0.03). Incongruent but not congruent objects yielded a significantly higher drop error for task‐irrelevant as compared to task‐relevant objects (interaction effect: *F*_(1, 1380.6)_ = 49.605, *p* < 0.001, *f* ^2^ = 0.04; congruent objects: *t*_(1420)_ = 0.241, *p*_*Holm*_ = 0.995, incongruent objects: *t*_(1412)_ = 9.559, *p*_*Holm*_ < 0.001). That is, spatial retrieval accuracy was comparable among congruently encountered objects, but for incongruently encountered objects, task‐irrelevant objects were spatially sorted less close to the actual location than task‐relevant objects. There was no difference between the two groups (*F*_(1, 1380.9)_ = 207.013, *p* < 0.001, *f* ^2^ = 0.15).

A semantic bias was estimated from the data of the SRT by determining in which room an incongruent object has been spatially retrieved, and calculating the proportion to which objects were sorted to the episodically fitting, semantically fitting, or unrelated room. We were able to replicate our previous findings: 1 day after encoding, participants sorted an incongruently experienced object significantly more likely to its semantically fitting room rather than to the unrelated room (*V* = 1192.5, *p*_*Holm*_ < 0.001). This was not true for task‐relevant objects (*V* = 388.5, *p*_*Holm*_ = 0.989). We hypothesized that cortisol behaviorally increases the semantic bias in memory retrieval. However, we found the same room sorting tendencies in both the cortisol and the placebo group: while the proportions of semantically sorted objects were significantly higher as compared to unrelatedly sorted objects for task‐irrelevant objects (cortisol group: *V* = 285, *p*_*Holm*_ < 0.001; placebo group: *V* = 328.5, *p*_*Holm*_ < 0.05), there was no such difference for task‐relevant objects (cortisol group: V = 108, *p*_*Holm*_ = 1; placebo group: *V* = 89.5, *p*_*Holm*_ = 1; in Figure 3B). Thus, cortisol did not lead to a behaviorally increased semantic bias in memory retrieval, though we still find an overall semantic bias in our paradigm (see Figure 3B).

### 3.3 Higher Similarity for Objects Belonging to the Same Semantic Category Pre‐Encoding

**FIGURE 4:**
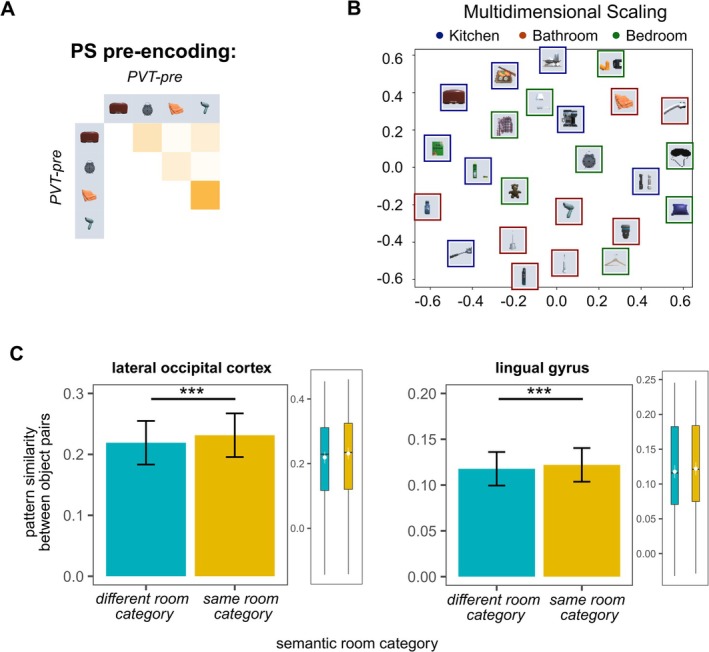
Pattern similarity pre‐encoding for same versus different room category object pairs in the lateral occipital cortex (LOC) and the lingual gyrus. (A) Schematic overview of pattern similarity (PS) pre‐encoding analyses. Object pairs are either from the same or from different semantic categories. We estimate pattern similarity for each object pair. (B) Multidimensional scaling visualizing PS in the LOC between all objects pre‐encoding reduced to two dimensions across participants. Category affiliation has been color‐coded. (C) Mixed effect model analyses controlling for random intercepts of participants found a significant main effect of semantic room category in the LOC and the lingual gyrus. In addition to the bar plots, visualizing averages, we provide estimated marginal means and confidence limits (white color), and boxplots showing the range of the underlying data. ***p < 0.001.

Pre‐encoding, we expected pattern similarity to reflect the semantic relation between objects: objects belonging to the same semantic room category should be more similar to each other as compared to objects belonging to different semantic room categories in regions of the brain associated with semantic knowledge representation. Indeed, we found this effect in the ventral visual stream (VVS, *F*_(1, 15,675)_ = 41.713, *p*_*Holm*_ < 0.001, *f* ^2^ = 0.002), specifically the LOC (*F*_(1, 15,675)_ = 65.344, *p*_*Holm*_ < 0.001, *f* ^2^ = 0.004; Figure 4C) and the lingual gyrus (*F*_(1, 15,675)_ = 13.077, *p*_*Holm*_ < 0.001, *f* ^2^ = 0.004). We also used multidimensional scaling to visualize the proximity of same and different category objects (see Figure 4B). Analyses were based on a total of 15,732 object pairs (276 object pairs for 57 participants). Full results tables for analyzed ROIs are included in Supporting Information S6.

### 3.4 Pattern Similarity of Incongruent Objects Post‐Encoding

**FIGURE 5:**
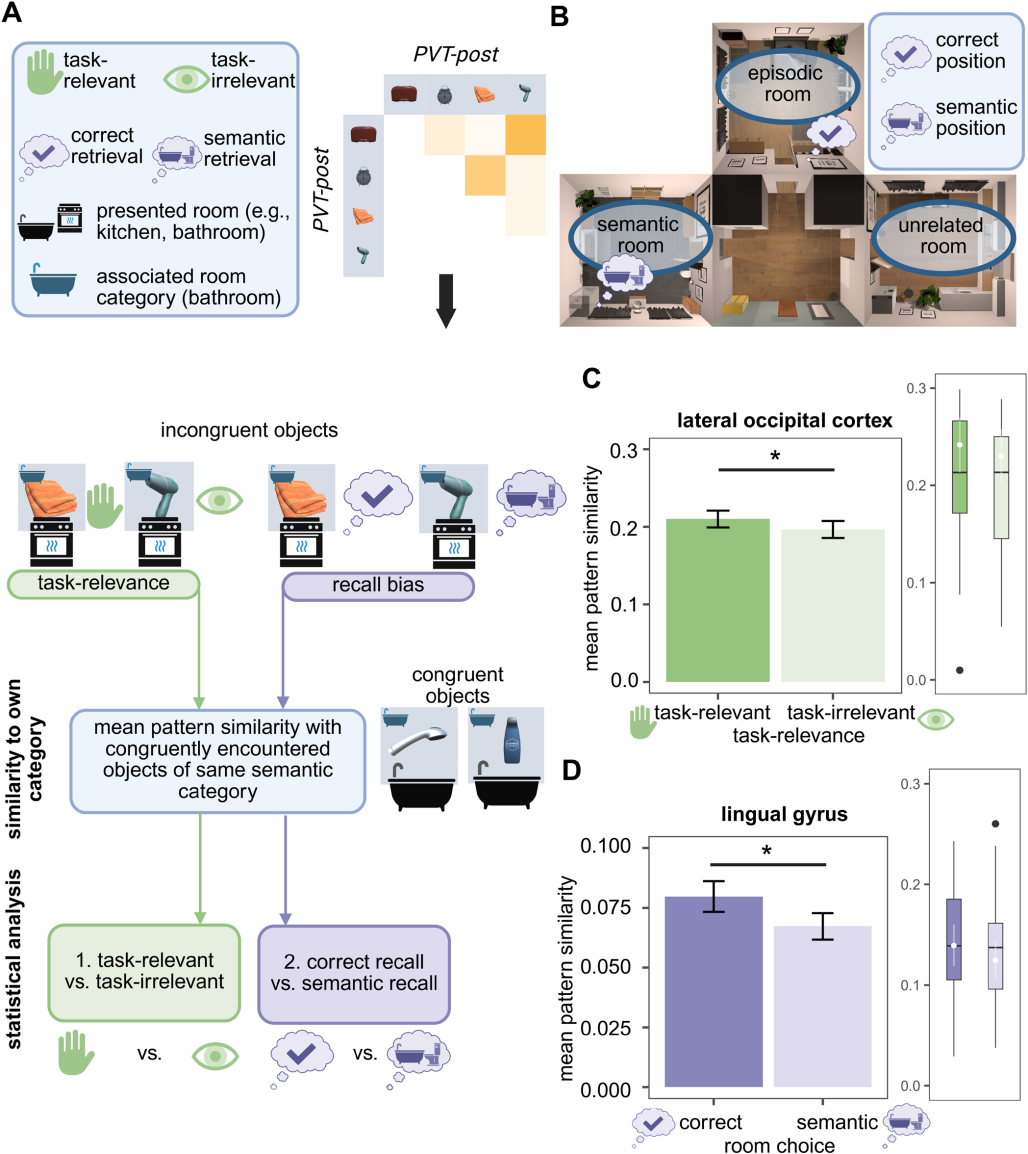
Visualization of analysis strategy and results obtained from analyses of mean similarity of incongruent objects to congruently encountered objects from the same semantic category post‐encoding. (A) Post‐encoding, we estimated mean pattern similarity of incongruent objects with congruently encountered objects of the same semantic category, and statistically compared resulting means between task‐relevant and task‐irrelevant objects and correctly sorted vs. semantically sorted objects. (B) Room choice options relevant for analyses: A correct choice refers to an incongruent object being retrieved in the room, in which it was actually encountered (episodic room), and a semantic choice indicates the retrieval of the spatial position in the semantically related, but incorrect room. (C) Significant main effect of higher mean pattern similarity to congruent objects of the same semantic category among task‐relevant objects compared to task‐irrelevant objects in the LOC. (D) Significant main effect of higher mean pattern similarity to congruent objects of the same semantic category among correctly sorted compared to semantically sorted objects in the lingual gyrus. In addition to the bar plots, visualizing averages, we provide estimated marginal means and confidence limits (white color), and boxplots showing the range of the underlying data. *p < 0.05.

We analyzed the semantic representation of an object post‐encoding, thus looking at neural pattern similarity of incongruent objects to congruently encountered objects from the same category (see Figure 5A). Supporting Information S7 includes results for all estimated models in these analyses.

*Task relevance*: In the LOC but no other included ROI (lingual gyrus, aHC, and pHC) we found a main effect of task relevance predicting pattern similarity (*F*(1, 52) = 7.77, *p*_*Holm*_ < 0.05, pes = 0.130). That is, the mean pattern similarity of incongruent objects to their congruent semantic counterparts was higher for task‐relevant objects compared to task‐irrelevant objects (see Figure 5C).

*Room‐placement*: We analyzed whether mean pattern similarity of incongruent objects to their semantically fitting category counterparts was associated with differences in room‐sorting, that is, comparing pattern similarity of objects sorted to the correct room and objects sorted to the semantically fitting room. We found a main effect of room‐sorting in the lingual gyrus (*F*(1, 52) = 8.56, *p*_*Holm*_ < 0.05, pes = 0.141), but not in the LOC or the hippocampus (all *p*_*Holm*_ > 0.05). In the lingual gyrus, mean pattern similarity of incongruent objects to their congruent semantic counterparts was higher when sorted correctly as compared to when they were sorted semantically (see Figure 5D).

### 3.5 Representational Change Was Predicted by the Retrieved Location and Retrieval Confidence

**FIGURE 6:**
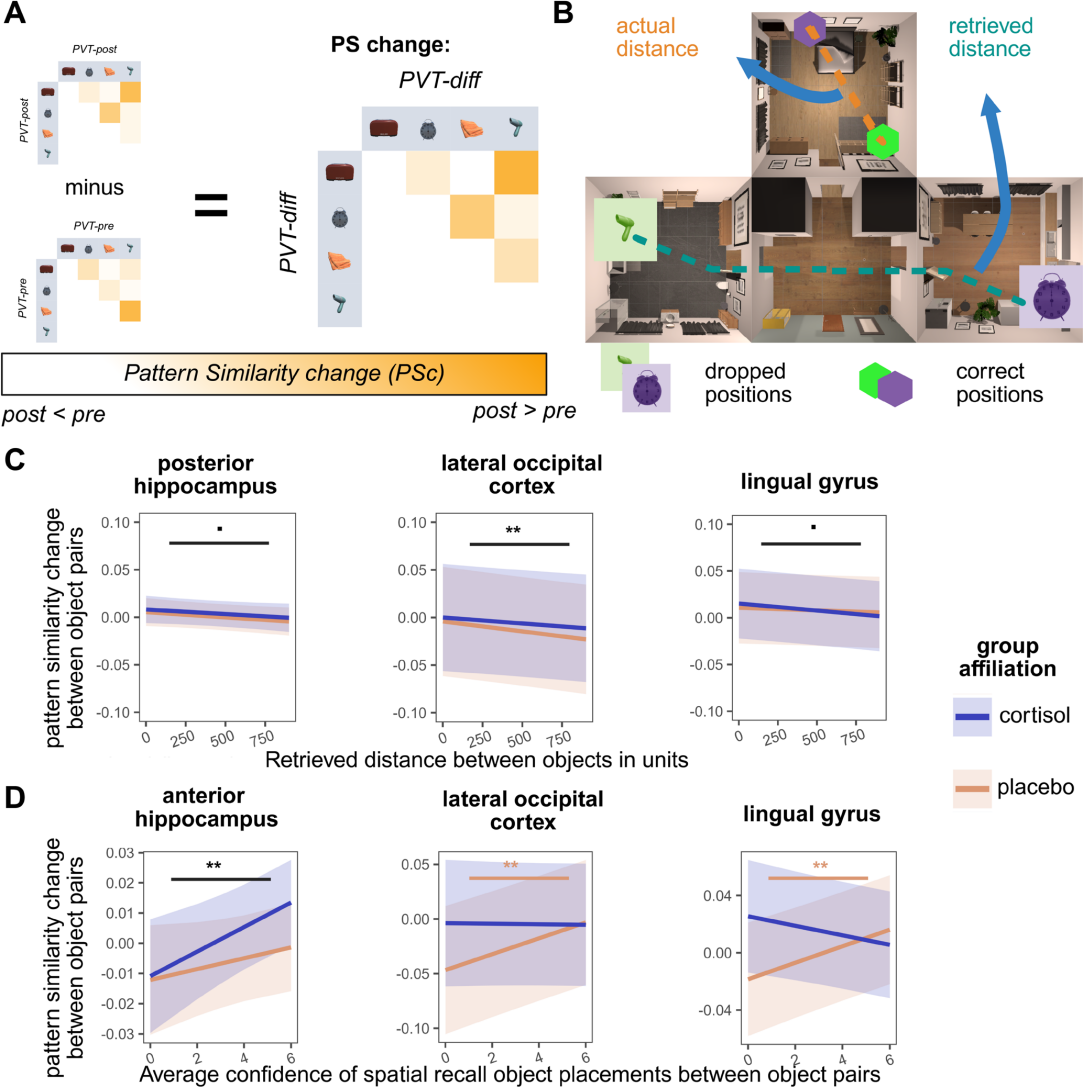
Visualization of results obtained from analyses of pattern similarity change. (A) Visualization of analysis strategy. Anal (B) Visualization of predictors: Retrieved distance between objects (green), actual distance between objects (orange) and calculation of distance error. (C) Retrieved distance between two objects predicting pattern similarity change in the pHC, the lingual gyrus and the LOC. We find a lower pattern similarity for higher retrieved distances in the LOC, and a similar trend effect in the pHC and the lingual gyrus. (D) Average confidence of spatial recall between objects as a subjective evaluation of memory accuracy, predicting pattern similarity change. We find a higher confidence to come along with higher pattern similarity change in the aHC, and, for the placebo group, in the LOC and the lingual gyrus. *p* < 0.1, *p < 0.05, **p < 0.01.

To shed light on the representation of encoded information, we related behavioral retrieval accuracy to the representational structure post‐encoding. Specifically, we estimated the difference value between pattern similarity post‐encoding and pre‐encoding to then analyze the representational change, that is, how pattern similarity between two objects has changed post‐ compared to pre‐encoding (see Figure 6A). As in behavioral analyses on a trial level, we excluded all object pairs for which at least one has not been recognized as “old” in the recognition task in order to exclude objects which might have not been seen at all in the virtual environment. This resulted in a number of 8411 object pairs from 51 participants included in the analysis, with an average of 164.92 (SD = 52.57, min = 45, max = 253) object pairs per participant. Exhaustive statistical summary tables are included in Supporting Information S8.

*The retrieved location in SRT*: Analyses revealed no significant difference in pattern similarity change contrasting object pairs which were remembered in the same or in different rooms in all our ROIs (all *p*_*Holm*_ > 0.1), and we furthermore found no interaction between group affiliation and same or different room recall (all *p*_*Holm*_ > 0.1). However, as a more fine‐grained measure we tested for a significant influence of retrieved proximity of two objects in spatial recall on pattern similarity change (as calculated by the distance between two objects placed in SRT, see Figure 6B). Interestingly, in the LOC, a higher retrieved distance between two objects was accompanied by a decrease in pattern similarity (*F*_(1, 8361.2)_ = 10.019, *p*_*Holm*_ < 0.01, *f* ^2^ = 0.001). In the pHC and the lingual gyrus, retrieved distance between two objects tended to predict pattern similarity change, though both effects did not survive the correction for multiple comparisons (pHC: *F*_(1, 8375.5)_ = 4.825, *p* < 0.05, *p*_*Holm*_ = 0.066, *f* ^2^ < 0.001; lingual gyrus: *F*_(1, 8362)_ = 5.231, *p* < 0.05, *p*_*Holm*_ = 0.066, *f* ^2^ < 0.001). A smaller retrieved distance between two objects tended to be associated with a positive pattern similarity change between two objects. In both regions, we did not find a significant interaction between group affiliation and proximity (all *p* > 0.05). In aHC, pattern similarity change was not associated with the retrieved proximity of objects in SRT (all *p*_Holm_ > 0.1). Results are depicted in Figure 6C.

*Confidence of recall in SRT*: During spatial recall of each object, participants indicated their confidence with regard to the location where they remembered having encountered it. For each object pair, we averaged the confidence in SRT and analyzed whether a higher confidence (i.e., self‐evaluation of memory accuracy) was associated with pattern similarity change in our ROIs. In the aHC, a lower average confidence significantly predicted a lower pattern similarity change (*F*_(1, 8291.1)_ = 10.360, *p*_*Holm*_ < 0.01, *f* ^2^ = 0.001). That is, the neural similarity pattern between two objects was more distinct post‐encoding if the objects were spatially sorted with a low confidence. There was no significant interaction of confidence and group affiliation (*F*_(1, 8291.1)_ = 1.551, *p*_*Holm*_ = 0.213, *f* ^2^ < 0.001). Similarly, analyses in the LOC showed a comparable pattern: a lower confidence was associated with a smaller pattern similarity change (*F*_(1, 8379.3)_ = 10.743, *p*_*Holm*_ < 0.01, *f* ^2^ = 0.001). Interestingly, analyses furthermore revealed a significant interaction effect of group affiliation with average confidence in the LOC and the lingual gyrus (LOC: *F*_(1, 8379.3)_ = 12.344, *p*_*Holm*_ < 0.01, *f* ^2^ < 0.001; lingual: *F*_(1, 8389.1)_ = 25.144, *p*_*Holm*_ < 0.001, *f* ^2^ = 0.003): While the cortisol group showed no association between confidence and pattern similarity change, a higher confidence was associated with a higher pattern similarity change in the placebo group. Finally, there was no significant effect in analyses of the pHC (all *p*_Holm_ > 0.1). Results are depicted in Figure 6D.

### 3.6 Reinstatement Effects for Incongruently Encountered Objects and Cortisol

**FIGURE 7:**
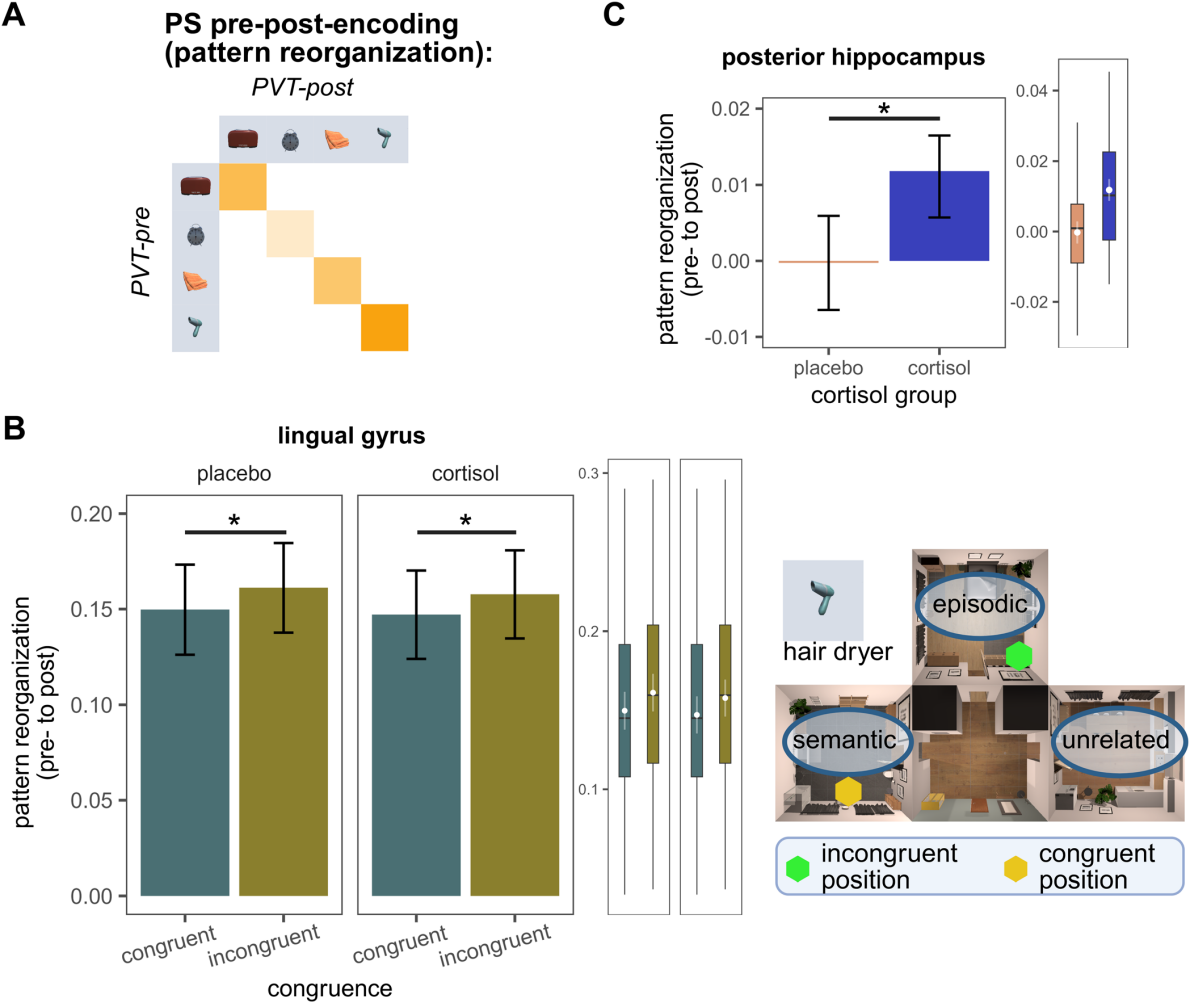
Visualization of results obtained from analyses of an object's pattern reorganization. (A) Visualization of analysis strategy. (B) Depiction of congruence. Pattern similarity for congruently and incongruently encountered objects in lingual gyrus. Bars indicate the model fit parameter of LMM analyses. Error bars depict the standard error. In lingual gyrus, incongruent objects show a lower pattern reorganization than congruently encountered objects. (C) Model fit parameters of cortisol group predicting pattern reorganization across participants in the pHC. Participants receiving cortisol showed a higher similarity compared to participants receiving a placebo. In addition to the bar plots, visualizing averages, we provide estimated marginal means and confidence limits (white color), and boxplots showing the range of the underlying data. *p < 0.05.

Additionally, we analyzed how similar an object was represented to itself pre‐ compared to post‐encoding, that is, pattern reorganization. We were able to relate memory performance (i.e., (1) correct room sorting, (2) distance between retrieved location to correct position, referred to as drop error, see Figure 7B and (3) confidence of spatial recall) and object characteristics (congruence; task relevance) to pattern reorganization (see Figure 7A). Group affiliation (cortisol vs. placebo) was included as a predictor in the models. Only recognized objects were included. Thus, from originally 24 objects per participant, these analyses were based on *M* = 18.41 (SD = 3.07, min. = 10, max. = 24) objects from 51 participants. Summary tables for statistical results are included in Supporting Information S9.

*Accuracy in SRT*: There was no significant effect of correct room placement on pattern reorganization of an object in any ROI included in our analyses (aHC, pHC, LOC and lingual, all *p*_*Holm*_ > 0.1). However, when looking at the distance between the retrieved location and the correct location (drop error), we found a trend toward a significant main effect in the aHC (*F*_(1, 892.32)_ = 5.184, *p* < 0.05, *p*_*Holm*_ = 0.081, *f* ^2^ = 0.009), and the lingual gyrus (*F*_(1, 896.26)_ = 5.409, *p* < 0.05, *p*_*Holm*_ = 0.081, *f* ^2^ = 0.006), however not surviving corrections for multiple comparisons. See Supporting Information S10 for detailed results and elaboration.

*Confidence of recall in SRT*: We found no significant association between the confidence of the spatial recall of an object and the pattern reorganization (all *p*_*Holm*_ > 0.1). In the model predicting pattern reorganization in the pHC, further investigations revealed a moderate variance inflation factor (> 5) for cortisol group affiliation and the interaction between the two. This indicates shared variance between the two predictors and thus limits the stability of this regression model. When including confidence as a single predictor for pattern reorganization in the pHC, there was still no significant effect (*p*_*Holm*_ > 0.1).

*Object characteristics*: In our analyses, objects that were encountered incongruently showed a higher pattern reorganization than objects which were encountered congruently during the EVE task in the lingual gyrus (*F*_(1, 888.78)_ = 9.079, *p*_*Holm*_ < 0.05, *f* ^2^ = 0.01, see Figure 7B), but not the aHC, the pHC, or the LOC (all *p*_*Holm*_ > 0.1). There was neither an interaction effect between group and congruence (all *p*_*Holm*_ > 0.1), nor a significant difference between task‐relevant and task‐irrelevant objects in any of our ROIs (all *p*_*Holm*_ > 0.1).

*Cortisol*: Interestingly, across three models (drop error, congruence, and task relevance), we observed a significant main effect of cortisol group affiliation on pattern similarity in the pHC, indicating that participants in the cortisol group exhibited systematically higher pattern reorganization compared to the placebo group. The significant main effect of group affiliation persisted only in models where specific predictors (e.g., drop error but not correctness of room placement) were included. Thus, we analyzed cortisol group affiliation predicting pattern reorganization in the pHC without other predictors and found a significant effect (*F*_(1, 51.345)_ = 7.434, *p* < 0.01, *f* ^2^ = 0.14, see Figure 7C).

### 3.7 Searchlight Analyses Reveal Distributed Networks Involved in Episodic Memory and Semantic Bias

**FIGURE 8:**
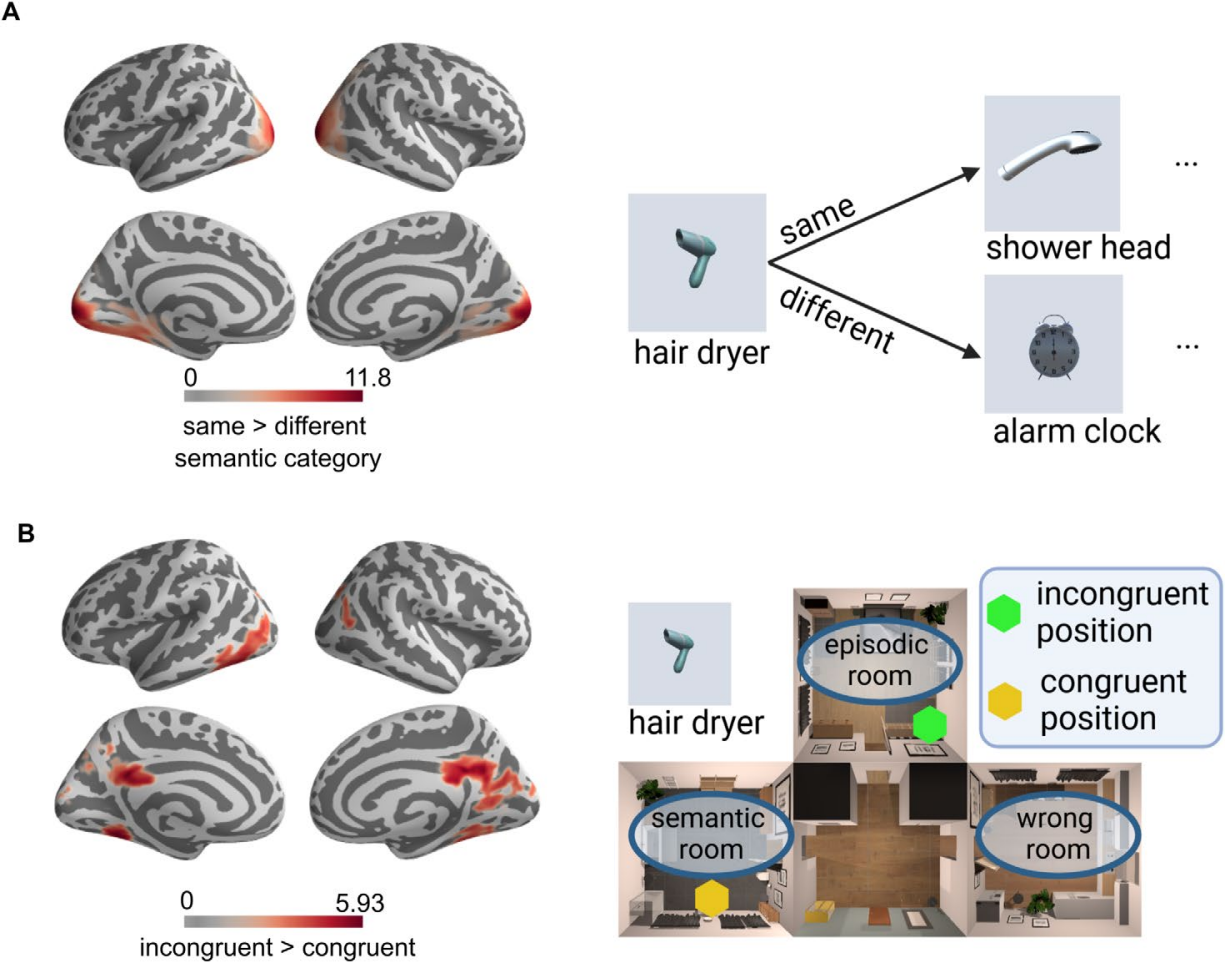
Depiction of significant clusters in searchlight analysis. (A) A visualization of significant clusters in which t‐values across participants were significantly different from 0 when estimating whether there is a significant difference in activation patterns between objects belonging to the same room category as compared to different room categories in each searchlight sphere. (B) Clusters in which there was a significant difference in mean activation patterns between objects encountered congruently during encoding as compared to objects encountered incongruently, estimated with a paired *t*‐test.

Pre‐encoding, the whole‐brain searchlight analysis confirms higher similarity for objects belonging to the same semantic category as compared to objects belonging to different semantic categories in the occipital cortex, specifically a cluster including the lateral occipital cortex, occipital fusiform gyrus, temporal occipital fusiform gyrus, lingual gyrus, posterior parahippocampal gyrus, and the occipital pole (*t*(50) = 11.8, *p*_*corr*_ < 0.01, FWE corrected using TFCE, see Figure 8A).

The within‐subject *t*‐test contrasting an object's pattern reorganization showed higher similarity for incongruently encountered as compared to congruently encountered objects, both in occipital regions and medial temporal lobe, specifically an extended cluster including the temporal occipital part of inferior temporal gyrus and middle temporal gyrus, intracalcarine cortex, posterior cingulate gyrus, precuneus cortex, cuneal cortex, lingual gyrus, temporal fusiform cortex, temporal occipital fusiform cortex, occipital fusiform gyrus, and supracalcarine cortex (*t*(50) = 5.93, *p*_*corr*_ < 0.01, FWE corrected using TFCE, see Figure 8B). Furthermore, task‐irrelevant objects showed a lower pattern reorganization compared to task‐relevant objects in the occipital pole (*t*(50) = 5.65, *p*_*corr*_ < 0.05, FWE corrected using TFCE). Looking at the spatial recall accuracy (correct room versus incorrect room recall), we found a small cluster of a higher similarity for correctly placed objects as compared to incorrectly placed objects (*t*(50) = 4.04, *p*_*corr*_ < 0.05, FWE corrected using TFCE) in the intracalcarine cortex.

## 4 Discussion

What influences whether we remember an episode or rely on prior knowledge to reconstruct past experiences? What will be the neural underpinnings leading to a shift from real episodic memory traces to the recruitment of a priori semantic knowledge? By creating conflict between an encoded episode and a priori semantic information, we wanted to investigate which memory system contributes to the retrieval of a past episode. Further, we aimed to shed light on how the neural representation of objects central (i.e., task‐relevant) to the encoding task (i.e., the gist) changed as a consequence of the encoded episode. To this end, we designed a virtual apartment and had participants conduct a series of actions with objects. Some of these objects were located in unexpected places. Furthermore, we administered cortisol to half of our participants prior to retrieval to impair episodic memory retrieval (Wolf 2017). We expected to find higher error rates and consequently a higher contribution of the semantic memory system in the cortisol group.

On a behavioral level, we were able to replicate our previous findings: for task‐irrelevant objects encountered in the virtual apartment, we found a semantic bias. Erroneously retrieved object locations tended to be retrieved according to the semantically fitting location. Also, congruent objects were retrieved more accurately than incongruent objects in our long‐term memory task. This is in contrast to findings from prediction error research, showing better memory for incongruent objects within scenes. However, there are multiple detrimental factors influencing whether there is a congruency or incongruency advantage, such as saliency, the interval between encoding and retrieval, or the number of incongruent objects (Allegretti et al. 2025; Frank et al. 2018; Greve et al. 2019). Despite a robust effect in previous studies showing that an increase in cortisol concentrations can impair retrieval (Kirschbaum et al. 1996; Schilling et al. 2013; Tollenaar et al. 2009; Wolf 2017), we found that cortisol did not impair the accuracy of episodic memory. It has been argued that specifically hippocampus‐based episodic retrieval (i.e., spatial recall) would be impaired as a consequence of cortisol increases, due to a high glucocorticoid and mineralocorticoid receptor density (Wolf 2017). One main hypothesis was that while cortisol impairs behavioral retrieval, the consolidated memory and respective neural pattern would be unaffected. This should be reflected in a higher semantic bias in the cortisol group. One explanation for our behavioral null findings could be the use of our encoding material: many studies administering cortisol use artificial behavioral paradigms for encoding (i.e., word lists or associations between two stimuli) (Antypa et al. 2022; Schilling et al. 2013; Schwabe and Wolf 2014). To our knowledge, there is no study investigating cortisol effects on spatial episodic memory after the encoding of a highly realistic episode. The episode included not only items relevant to our analysis but also many further details, decorations, and a realistic context. The gist‐retrieval of a complex, contextually embedded episode might function differently as compared to the retrieval of word lists (Shields et al. 2017). Thus, the retrieval of a complex scene might be less susceptible to cortisol effects. In our task, participants were confronted with a rich environment with plenty of cues to ease retrieval, especially in terms of cued recall. Accordingly, behavioral retrieval of task‐relevant objects in cued recall was very good in both groups. A quantification of retrieval beyond item memory, also considering other aspects of the encoded episode, might have revealed differences between the cortisol and the placebo group. To summarize, our results suggest a subtle and complex influence of cortisol on the reconstruction of a realistic past episode and the need for further investigations.

Next, we investigated the neural underpinnings of episodic versus semantically biased retrieval and how task relevance would influence this interplay between true and false memories. Specifically, for incongruent objects, we expected participants to employ prior knowledge in cases of uncertainty. Thus, we focused on the mean pattern similarity of an incongruent object to semantically related but congruently encountered objects (i.e., toothbrush in the kitchen vs. rubber duck, hairdryer in the bathroom) and statistically estimated whether there was a difference between (A) task‐relevant and task‐irrelevant objects and (B) correctly retrieved and semantically retrieved objects. Interestingly, we found that during retrieval, task‐relevant objects were, on average, more similar to their semantically related objects than task‐irrelevant objects in the LOC. In addition, correctly retrieved incongruent objects were likewise more similar to semantically related objects than objects that were retrieved to have been in the semantically associated room. Behaviorally, task‐relevant objects were retrieved more correctly compared to task‐irrelevant objects, which is in line with previous findings on the role of enactment and attention in memory (Brooks et al. 1999; Peterson and Mulligan 2010; Roberts et al. 2022). Thus, attentional mechanisms during encoding defined to what extent a semantic bias is present during retrieval. On a neural level, objects that were correctly retrieved showed, in turn, a higher similarity to objects from their semantic category. This might represent a strengthening of the original category representation of task‐relevant and correctly retrieved objects in cases of attentive, incongruent encounters. Overall, we found superior long‐term memory for congruent compared to incongruent objects. However, the previously presented findings highlight how incongruent encounters can lead to overt attention in some cases, possibly mediated by saliency (Allegretti et al. 2025).

Afterwards, we analyzed how neural pattern similarity changed following encoding and how this change in representations would relate to retrieval performance. We found incongruently experienced objects to show a lower pattern reorganization (in form of pre‐ to post‐encoding similarity) as compared to congruently encountered objects in the lingual gyrus, a region typically associated with semantic knowledge (Devereux et al. 2013). This might represent an interaction between the episodic memory system and prior knowledge: the representation of an object after a surprising encounter in an unexpected location seems to be strengthened on a neural level, creating a distinct episodic representation in cases of conflicting a priori information (Frank and Kafkas 2021; Ranganath and Rainer 2003). Thus, while a toaster would usually be placed in the kitchen, this specific toaster may be distinctly remembered to be placed in the bathroom, due to its strengthened memory representation.

When focusing on representational change between objects, we found unconfident object placements to be associated with a decrease in pattern similarity (post‐encoding < pre‐encoding similarity) in the aHC. This might represent a distinctiveness of objects which were recognized from encoding but not associated with a spatial location. This distinctiveness suggests a contextually not embedded gist‐memory of the episode, which was behaviorally retrieved with a higher involvement of semantic knowledge.

With regards to spatial associations between objects, we found a trend toward a similarity increase in the pHC for objects which have been retrieved as being spatially close to each other, irrespective of their temporal proximity (i.e., toaster and hairdryer on the kitchen table: using the toaster at the beginning of the sequence of action and using the hairdryer last). This strengthens the idea that the pHC is involved in the spatial associations between objects (Brunec et al. 2020). Importantly, our results show that the objects of the encoded episode (i.e., the gist of the tasks) were contextually bound together. The same pattern was found in the LOC and the lingual gyrus, which suggests a connection between hippocampal and neocortical areas involved in object and category representation. The involvement of the LOC and the lingual gyrus in contextual binding of semantically strongly categorized objects supports the idea that different brain regions support memory accessibility and representation by pattern similarity change in different dimensions. This has also been shown for emotionally relevant binding in the amygdala (Bierbrauer et al. 2021; Martin and Barense 2023). Furthermore, to adapt to the environment, categorical knowledge needs to be flexibly updated. It is unlikely that after one exposure to partly unexpected object combinations the representation of object pairs, formerly unrelated, would have changed fundamentally. However, our data might show the representation of categorical exceptions as a starting point of an updating of semantic knowledge in consequence to an association between objects (Duff et al. 2019). Interestingly, searchlight analyses did not reveal pattern similarity change or reinstatement in other neocortical brain regions, though a contribution of, for example, frontal regions is likely (Renoult et al. 2019; Robin and Moscovitch 2017). Theories suggest that the retrieval of schema congruent information is mediated by prefrontal structures as opposed to schema incongruent information (Audrain and McAndrews 2022; van Kesteren et al. 2012). However, the task demand, that is, the passive encoding of objects, as opposed to active retrieval, might have relied more on visual processing with less involvement of frontal regions.

Finally, we aimed to investigate the effects of cortisol on neural similarity and its link to memory performance. Despite not finding differences in behavioral analyses between the two groups, we found cortisol to cause effects on the neural level: First, looking at pattern reorganization of an object to itself, we found a lower reorganization in the cortisol group as compared to the placebo group in the pHC, independent of behavioral performance. This might hint toward a difference in (re‐)encoding in the cortisol group during this repeated exposure to relevant stimuli (Sherman et al. 2023); however, it did not result in differences in behavior when looking at the retrieval accuracy of the originally encoded episode. Future studies might shed light on the effect of cortisol on pattern reorganization. Analyses furthermore revealed group differences in the analyses of neural pattern similarity predicting behavioral retrieval. Analyzing representational change between objects, we found confidence in spatial recall to be associated with a higher pattern similarity change in the LOC and the lingual gyrus only for the placebo group but not for the cortisol group. First, this underlines the influence of cortisol on retrieval from extra‐hippocampal patterns despite a lower density of mineralocorticoid receptors (ter Heegde et al. 2015) and expands the small body of research on this topic (Wolf 2017). Second, this finding indicates the presence of a divergence between neural representations and behavioral retrieval confidence as a consequence of cortisol intake. As argued before, increasing the number of stimuli or a quantification of retrieval beyond item‐memory in the present task would increase the sensitivity to detect possible differences in behavior between the two groups. Future studies are needed to elucidate the association between heightened cortisol levels and neural pattern similarity.

Our results are promising on multiple levels. However, it remains challenging to obtain an optimal timing of tasks in study designs: First, by exposing participants to the PVT prior to encoding, all objects included in the episode were already seen multiple times in a random order. Although balanced across all objects, this prior exposure has likely influenced contextual encoding. Conversely, the second PVT might have in turn been influenced by the memory tasks timed before. Our results are still applicable to current models of neural representational patterns, and previous studies have also used passive encoding of objects to investigate neural pattern similarity with respect to memory performance of a past episode (Bierbrauer et al. 2021; Deuker et al. 2016). However, one could argue that the specific analysis of pattern similarity during active instead of passive encoding has its advantages as well, considering the importance of task demand on representational patterns (Brunec et al. 2020; Renoult et al. 2019). Another point of consideration lies in the timing of cortisol application. As cortisol should influence retrieval accuracy, we administered cortisol in a way that participants had increased cortisol levels throughout the whole testing day, during which the retrieval tasks and the second PVT took place. While the impairing influence of acutely increased cortisol concentration on memory retrieval has been elucidated, it has not been subject to intense investigation how it influences neural representational patterns (Bierbrauer et al. 2021). In future studies one might consider conducting the post‐encoding PVT when cortisol levels are equal between groups (i.e., prior to retrieval or after retrieval), to be able to clearly attribute associations between behavioral and neural responses to the influence of cortisol on behavioral response patterns and avoiding the influence of cortisol on neural responses. Our observed effect sizes, while small, are comparable to those reported in previous studies examining representational similarities (Bellmund et al. 2022; Bierbrauer et al. 2021). Hence, our effects should be interpreted in the context of the sensitivity of RSA in detecting subtle, but meaningful, differences in neural representations. Of note, reasons for not replicating all results obtained from ROI analyses in our searchlight analyses might lie in (a) the necessity for more extended corrections for multiple comparisons in whole‐brain analyses, and (b) the difference in analysis strategies: while we used LMMs for ROI analyses, we obtained t‐maps (estimating differences in pattern similarity between dichotomous predictors within each searchlight) or correlation maps (estimating correlations between continuous predictors for object pairs and pattern similarity) covering the whole brain and then tested for significant clusters, or, in a second approach, contrasted searchlight maps for the two levels of our dichotomous predictors. Importantly, in our searchlight analysis, we did not statistically consider clustering in the data for individual participants (i.e., by using a multilevel approach). One major caveat comes down to our sample being limited to only male participants: the exclusion of female participants limits the generalizability of our findings to male populations. While hormonal fluctuations that naturally occur in the female cycle and their interplay with cortisol potentially influence the semantic bias, our study design did not account for this variability. Since our study was aimed to be a first step into understanding the neural correlates of semantic bias during retrieval and the role of cortisol in increasing this bias, we chose to focus on male participants to keep the already complex study design feasible. Future studies should investigate the role of cortisol and gender on semantic biases using a more representative sample of both males and females. Additionally, future research could explore how menstrual cycles and hormonal use affect semantic processing specifically in females, which may provide insights into the neural basis of these biases that potentially occur naturally in the course of the menstrual cycle in women without direct cortisol intake.

## 5 Conclusion

Making use of a highly standardized yet realistic encoding environment, we were able to demonstrate how increased semantic representation of incongruent objects leads to better memory. With the creation of a conflict between episodic and semantic information, we found strengthened memory traces for exceptional objects suggestive of reinstatement, which is furthermore reflected in an effect of cortisol on a neural level. Overall, our findings on incongruent objects suggest an influence of prior semantic knowledge on the construction of past episodes, reflected in neural similarity patterns.

## Ethics Statement

The study was approved by the local medical ethic committee of the Ruhr University Bochum, Reg.‐No. 18‐6368 and was conducted in accordance with the Declaration of Helsinki.

## Consent

All participants gave informed consent to participation.

## Conflicts of Interest

The authors declare no conflicts of interest.

## Supporting information


**Data S1:** Supporting Information.

## Data Availability

The data that support the findings of this study are openly available in osf at https://osf.io/5k3mx/.
