# Supplementary material for: A Toaster in the Bathroom: Neural Correlates of Semantic Construction During Episodic Memory Recall
Source: Hum Brain Mapp. 2025 Oct 25;46(16):e70389. doi: 10.1002/hbm.70389 (PMC12553115; doi:10.1002/hbm.70389)
Supplement: Supplementary file 1 — Data S1: Supporting Information. [file HBM-46-e70389-s001.docx]

**Supplemental Material**

**A toaster in the bathroom: Neural correlates of semantic construction during episodic memory recall**

Carina Zoellner^1*^, Rebekka Heinen^2*^, Nicole Klein^1^, Nora A. Herweg^2^, Christian J. Merz^1^, Oliver T. Wolf^1^

^1^Cognitive Psychology, Institute of Cognitive Neuroscience, Faculty of Psychology, Ruhr University Bochum, Bochum, Germany

^2^Department for Neuropsychology, Institute of Cognitive Neuroscience, Faculty of Psychology, Ruhr University Bochum, Bochum, Germany

*Both authors contributed equally to this publication

## S1. Materials

*Stimuli – validation of category affiliation*

In a small piloting with independent raters (*N* = 8) we verified that objects were sorted to the category we initially intended the objects to be affiliated with. To this end, participants were presented with an depiction of this object and a room-label (either kitchen, bathroom or bedroom) and asked to rate on a scale from 1 to 7 whether they would typically expect this object in that room. Thus, each object was presented 3 times (72 trials per participant). Figure S1 includes a visualization for each object and category separately.

**Figure S1**

Validation of stimuli with *N* = 8 participants

A
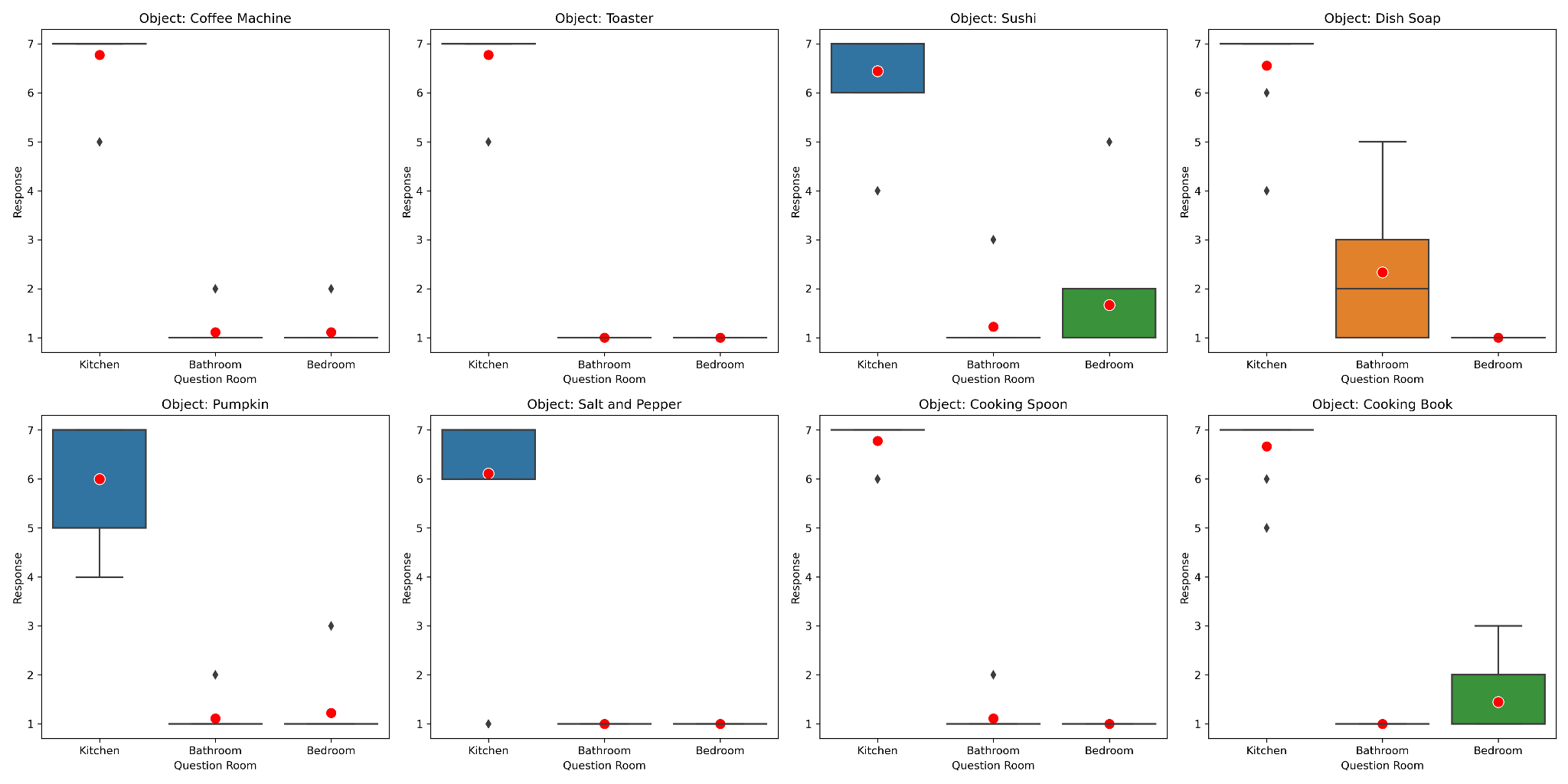
B
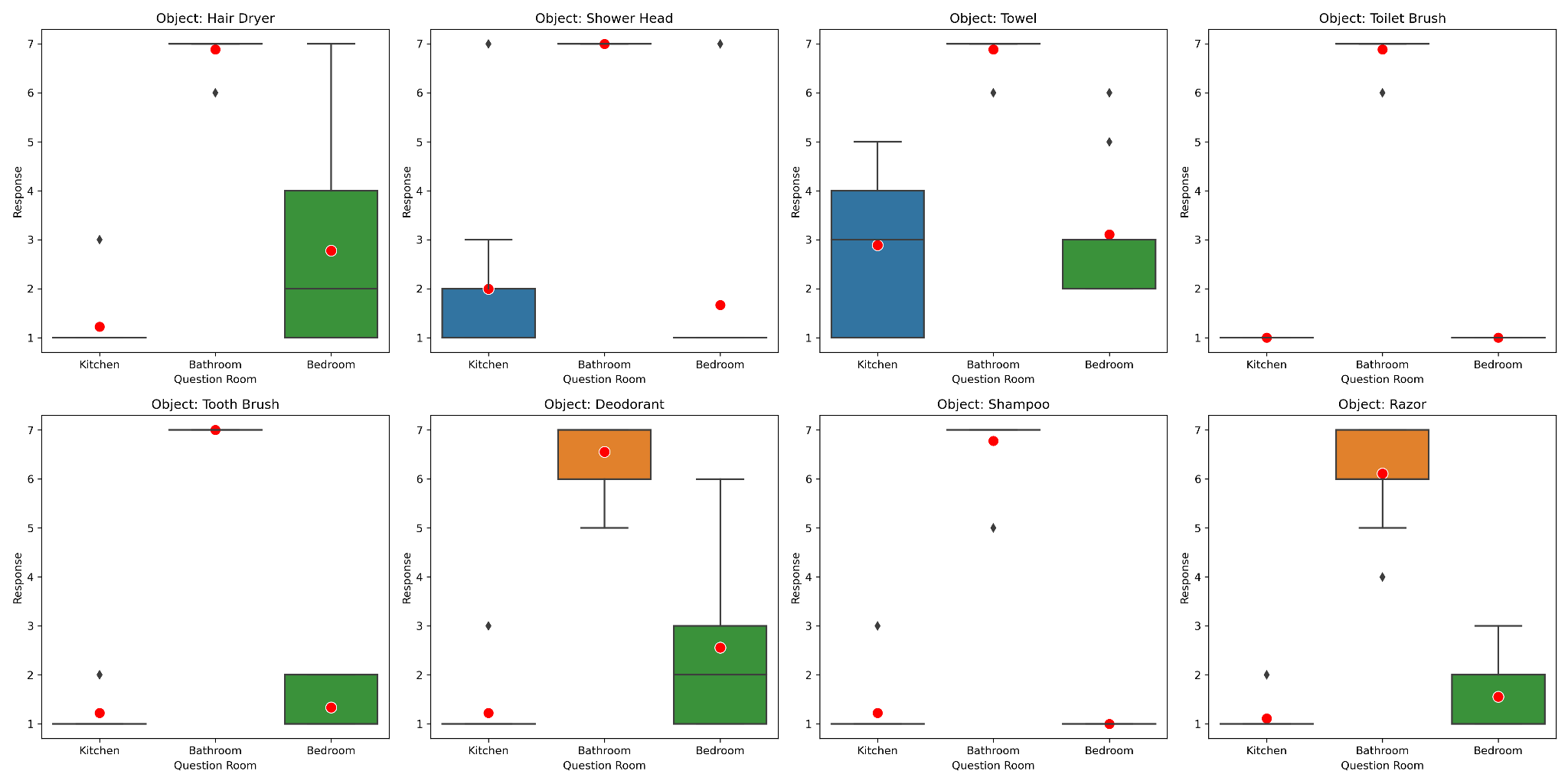
C
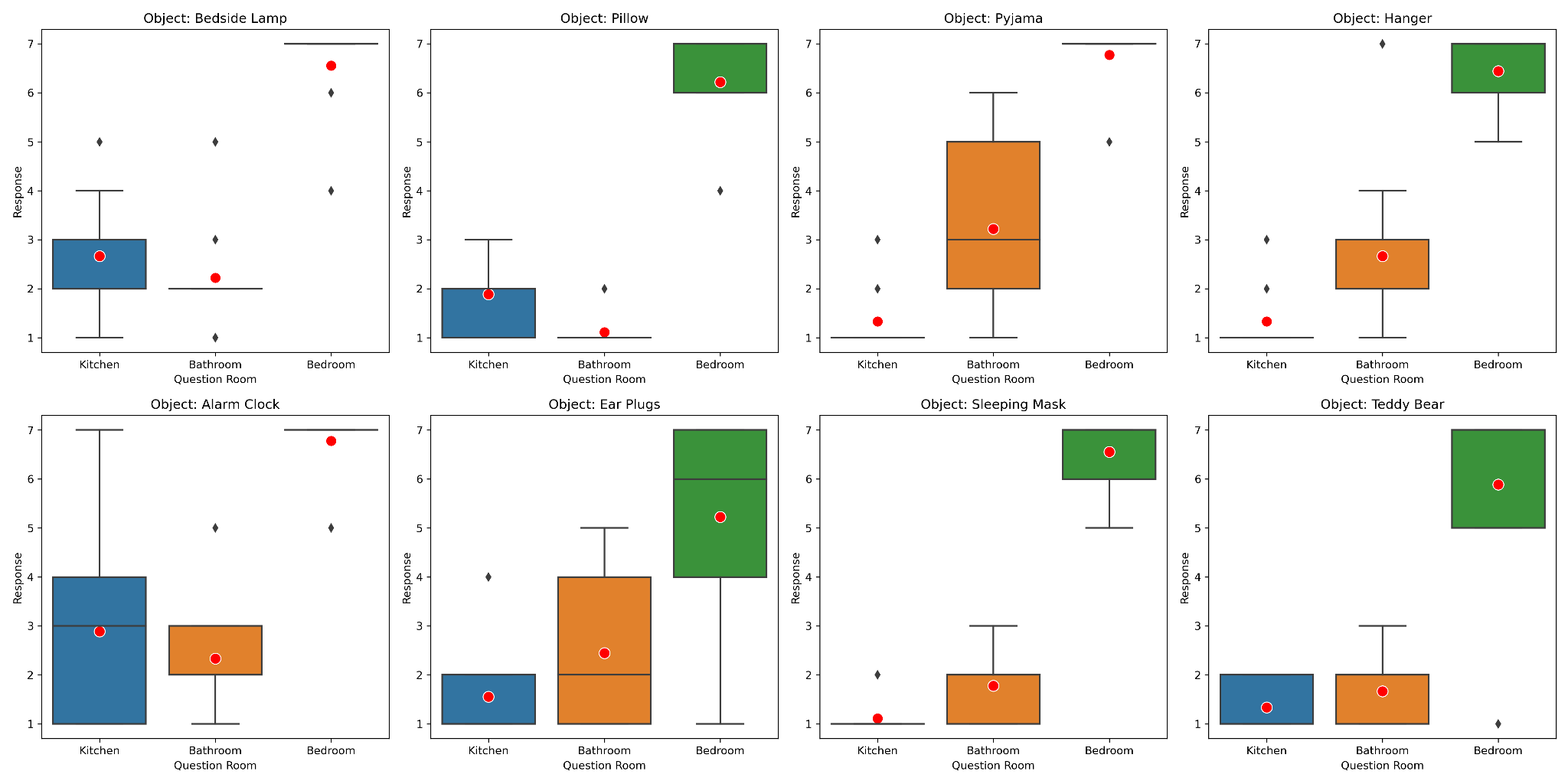


*Note. Depicted are boxplots showing median, standard deviation, error bars and outliers for ratings of objects to the respective room category. Additionally, red dots indicate mean values. A includes objects originally chosen for the kitchen, B includes objects originally sorted to the bathroom and C includes objects originally chosen for the bedroom.*

We summarized that objects were overall associated with the room we previously associated it with. Note that one kitchen object (pumpkin) was later replaced by a non-food object (drying rack) to increase comparability between category and have the focus on objects inviting to perform an action. This new object was not validated separately.

*Tasks*

Tasks within the encoding paradigm were centered around an object, and always included the participant to 1) look for an object, 2) approach it and 3) interact with it. Subsequent to an interaction, the object changed slightly. This change could be of visual or spatial nature (i.e., the coffee machine was full instead of empty, or the hairdryer appeared in a slightly different spatial orientation and location after interaction).

**Table S1**

*Objects and associated tasks.*

| ***Object number*** | ***Object name*** | ***Room*** | ***Object task*** |
| --- | --- | --- | --- |
| *Object 01* | *coffee machine* | *kitchen* | *‘Go to the coffee machine and make yourself a coffee.’* |
| *Object 02* | *toaster* | *kitchen* | *‘Go to the toaster and prepare yourself a sandwich as a quick snack.’* |
| *Object 03* | *dish rack* | *kitchen* | *-* |
| *Object 04* | *sushi plate* | *kitchen* | *-* |
| *Object 05* | *dish soap* | *kitchen* | *‘The dish soap leaked. Clean it up.’* |
| *Object 06* | *cooking book* | *kitchen* | *‘You still need to buy groceries. Look for the recipe inside the cooking book and take a picture of the list of ingredients.’* |
| *Object 07* | *salt and pepper grinder* | *kitchen* | *-* |
| *Object 08* | *spaghetti spoon* | *kitchen* | *-* |
| *Object 09* | *hair dryer* | *bathroom* | *‘After the shower your hair is still wet. Pick up the hair dryer to blow dry them.’* |
| *Object 10* | *shower head* | *bathroom* | *‘The broken showerhead is lying around your flat for ages. Go pick it up and finally throw it away.’* |
| *Object 11* | *towel* | *bathroom* | *-* |
| *Object 12* | *toilet brush* | *bathroom* | *-* |
| *Object 13* | *electric toothbrush* | *bathroom* | *‘Don't forget to charge your electric toothbrush.’* |
| *Object 14* | *deodorant* | *bathroom* | *‘You want to make a positive first impression. Don't forget to use deodorant.’* |
| *Object 15* | *shampoo* | *bathroom* | *-* |
| *Object 16* | *razor* | *bathroom* | *-* |
| *Object 17* | *bedside lamp* | *bedroom* | *‘Take care of the perfect atmosphere and switch on the bedside lamp.’* |
| *Object 18* | *pillow* | *bedroom* | *‘You still need to do the bedding. Start with the pillow.’* |
| *Object 19* | *pyjama* | *bedroom* | *-* |
| *Object 20* | *hanger* | *bedroom* | *-* |
| *Object 21* | *alarm clock* | *bedroom* | *‘Tomorrow you'd like to sleep in. Don't forget to set off the alarm clock.’* |
| *Object 22* | *earplugs* | *bedroom* | *‘Last night your neighbours have been very loud again. Your earplugs are still lying around. Put them back into their case.’* |
| *Object 23* | *sleeping mask* | *bedroom* | *-* |
| *Object 24* | *teddy bear* | *bedroom* | *-* |

*Picture Viewing Task*

Each of the four PVTs, two prior to encoding, two approximately 24 hours after encoding, consisted of six blocks, each containing 24 pictures showing the target objects from the encoding phase in random order. Every item was presented for 1500 ms, followed by an inter-trial interval of 3750 ms or 5750 ms (uniformly randomly distributed), during which a fixation cross was presented. To make sure participants paid attention to the objects we used an oddball cover task: 5% of all 144 pictures within one PVT were marked with a small picture of a fly for 400 ms. Participants were instructed to press a button with the index finger whenever they detected a fly using a MR-compatible button box. Blocks were separated by a short break of 30 seconds. Between the third and fourth block, the break was slightly longer (35 seconds), as feedback on the participants’ performance (i.e., how many flies were detected) was shown. The feedback was also presented at the end of the sixth block. The functional data from the PVT is the basis for the analysis of neural representational change. We excluded blocks in which more than two flies were missed because we could then not be sure that a participant actually paid attention to the objects presented during this task. The PVT was presented and programs using Presentation software (Version 22.1, Neurobehavioral Systems, Inc., Berkeley, CA, [www.neurobs.com](http://www.neurobs.com)).

*Free Recall*

In a recorded, oral free recall test, participants were asked to freely report everything they can recall from their experience within the virtual environment. After being handed a written instruction, the participants listened to an example of a recall based on the virtual example try-out task in order to get the participants’ focus on object- and task-recall. The example had a duration of 2:40 min. Participants were alone during their audio recording. The recorded audio file was transcribed and screened for objects by two independent raters, extracting the information if an object has been mentioned, in which room, and at what point in time. From this task, we can look at a) which objects were freely recalled, b) which rooms these objects were recalled in if an according location was named and c) in what order objects and tasks were recalled. We used the program Audacity for audio recording (v.3.2.1). In the online setting 28 days after encoding, the participants were asked to upload an anonymized, self-recorded audio file to a cloud-server using their generated individual subject code.

*Recognition Task*

During the recognition task, participants saw the same pictures of objects as in the PVT. In addition to the 24 target objects from the encoding phase (“old” objects), we included 24 lures, see figure S2, which were matched with regard to the likelihood to appear in a virtual housing environment. Each picture was presented for four seconds. Subsequent questions regarding each object were presented until a key response was registered. Responses were given via a MR-compatible button box with both hands. Participants rated the objects as old or new on a 6-scale rating, ranging from ‘sure old’ to ‘sure new’. If a participant referred to an object as old (i.e., seen in the virtual environment), they were also asked to indicate in which room they had encountered the object and their confidence of their room choice for this object. In addition to an analysis of the responses to individual objects, we calculated the response bias-corrected sensitivity measure *d’* to estimate the general recognition ability of each participant. The task was structured similarly in the online setting, only that there was no time-limit in the presentation of each object picture. The recognition task was programmed and presented using MATLAB (MathWorks, Version 2020b) psychtoolbox extension OTBR toolbox (Brainard, 1997; Otto & Rose, 2023). Responses were provided with a MR-compatible button box.

*Figure S2*

*Overview of distractor objects during recognition task.*

*
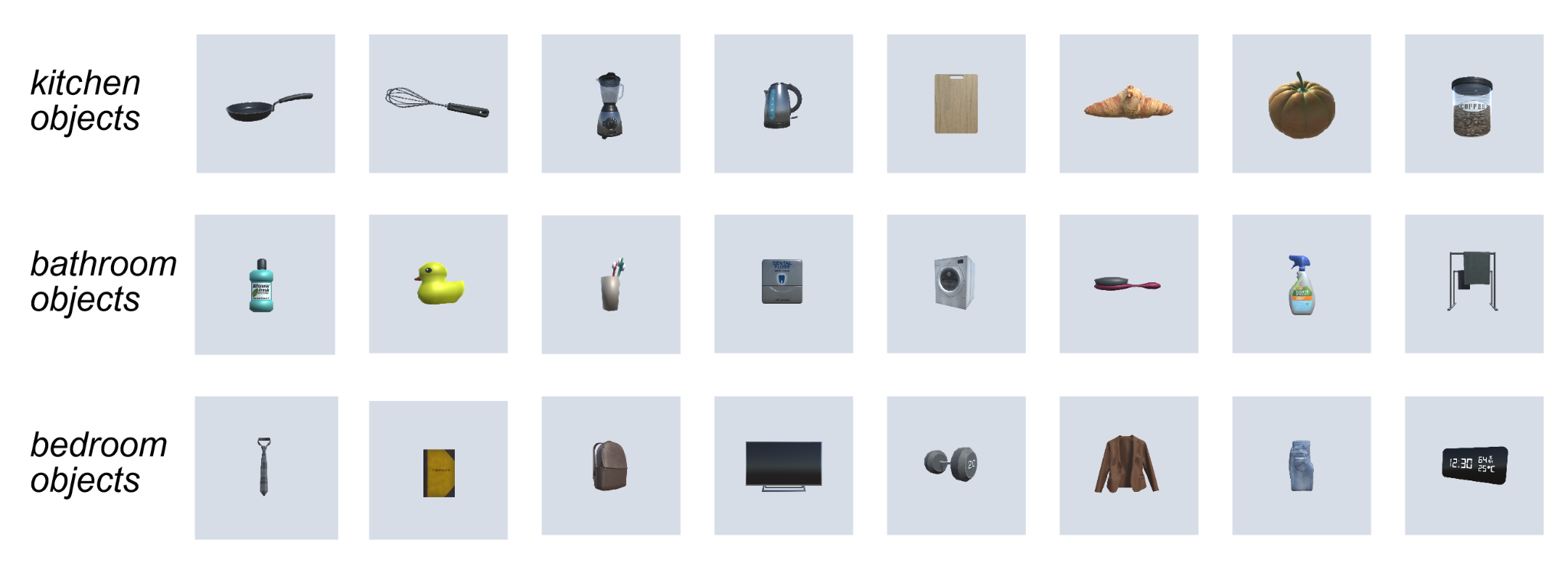
*

*Note. Lures were chosen to match one of the three categories (kitchen, bathroom, bedroom). kitchen: pan, whisk, mixer, water cooker (kitchen item frequently used in Germany), cutting board, croissant, pumpkin, coffee beans. bathroom: mouthwash, rubberduck, toothbrush, dental floss, washing machine, comb, cleaning agent, towel dryer. bedroom: tie (only male participants), diary, backpack, TV, barbell, jacket, jeans, digital alarm clock.*

*Spatial and Temporal Recall*

After fMRI measurements on the day after encoding, participants performed a spatial recall task (SRT) on a laptop in a preparation room at the hospital. During the SRT, participants saw the virtual environment form bird’s eye perspective and could drag objects to the location where they remembered to have encountered them during the VR task. In this task, the relative distance from the placed object to its original position in the virtual environment was measured (considering the shortest possible path between them; drop error). We additionally asked for the participants’ confidence on each of their object placements. Due to technical limitations, we did not administer the SRT in the online setting four weeks after encoding.

Directly following to the SRT, participants performed two temporal recall tasks (TRTs). During these tasks, participants sorted all objects according to their temporal occurrence. First, all task-relevant objects were shown and were draggable to numbered positions (1-12) representing the subjective memory of the temporal order of the tasks in the sequence of actions during the EVE task. On a next screen, all 24 objects were presented, and participants were instructed to reconstruct the order of their conscious encounter with the objects in the virtual environment, irrespective of whether they had interacted with an object or not. For both subtasks, the encoded and retrieved pairwise temporal distance as a measure of accuracy was calculated. Similarly, the semantic construction effect, i.e., whether objects belonging to the same semantic category are temporally clustered together during retrieval was analyzed. We additionally asked for the participants’ confidence on each of their object placements in the temporal order. In the online setting, the TRT was similar, but instead of dragging pictures of objects to numbers representing the positions in the temporal order, participants were asked to insert numbers next to pictures of objects to indicate their remembered place in the sequence of actions.

SRT and TRT were created and presented using Unity (Version 2019.1.3, Unity Technologies, San Francisco).

*Questionnaires*

Subsequent to the EVE on the first day of data collection, participants were asked to fill in the Igroup Presence Questionnaire (IPQ, Schubert et al., 2001) to assess the subjective experience in the virtual environment, measured on the dimensions spatial presence, experienced realism, involvement and one general item which correlates with all other dimensions. Additionally, in parallel to each collection of saliva samples, we measured the emotional state of a participants using the positive and negative affect schedule (PANAS, Breyer & Bluemke, 2016).

## S2. Detailed statistical results behavioral memory retrieval

**Free recall**

On average, each participant freely retrieved *M* = 8.47 objects (*SD* = 2.64). From those objects, *M* = 7.40 (*SD* = 2.57) were retrieved together with a room.

Of all objects across participants in free recall, 58.333 % of task-relevant objects and 12.222 % of task-irrelevant objects were retrieved. 75.714 % of all freely retrieved task-relevant objects were placed in the correct room, 4.523 % in the semantically fitting, 8.333 % in the unrelated room and 11.429 % were not sorted to a room at all. With regards to task-irrelevant objects, 63.636 % were sorted to the correct room, 9.091 % to the semantically fitting room, 7.955 % to the unrelated room and 19.318 % were not sorted to a room at all.

This was more balanced between congruent and incongruent sortings: overall, 33.889 % of congruent objects and 36.667 % of incongruent objects were freely retrieved. 82.378 % of congruently experienced items were placed in the correct room, 4.508 % in the unrelated room and 13.115 % were not sorted to a room at all; while 65.530 % of incongruently experienced objects were placed in the correct room, 10.227 % in the semantically fitting and 11.742 % in the unrelated room. 12.500 % of correctly retrieved incongruent objects were not sorted to a room at all.

In a logistic linear mixed model with subjects as random factors, we found that task-relevant objects were significantly more likely freely retrieved than task-irrelevant objects (OR = 0.096, 95% CI [0.060, 0.155]), while we did not find a main effect of cortisol group affiliation (OR = 1.093, 95% CI [0.703, 1.699]) or congruence (OR = 1.294, 95% CI [0.872, 1.920]), neither did we find an interaction effect between congruence and task-relevance (OR = 1.052, 95% CI [0.611, 1.812]), cortisol group affiliation and congruence (OR = 0.813, 95% CI [0.492, 1.344]) or group affiliation and task-relevance (OR = 0.878, 95% CI [0.509, 1.515]). With regards to correct room recall of all objects, analyses revealed a significant main effect of task-relevance (OR = 0.115, 95% CI [0.076, 0.174]), but again no effect of cortisol group affiliation (OR = 0.838, 95% CI [0.589, 1.194]), congruence (OR = 0.827, 95% CI [0.611, 1.119]), or an interaction effect, tested between congruence and task-relevance (OR = 0.725, 95% CI [0.385, 1.367]), cortisol group affiliation and congruence and group affiliation and task-relevance.

To summarize, on the one hand, participants could well remember objects which were part of the sequence of action. This was also the strongest predictor for a related correct retrieval of the room in which the object was encountered. On the other hand, free recall of an object was not influenced by whether an object was encountered congruently or incongruently.

**Recognition task**

Overall, participants had a hit rate of *M* = 0.77 (*SD* = 0.13), that is, they correctly identified an object as old in 77 % of the cases. Dee Prime as a measure for recognition-memory was on average *M* = 1.69 (*SD* = 0.83). We did not find a significant difference of deePrime between the two groups (cortisol group: *M* = 1.577, *SD* = 0.87, placebo group: *M* = 1.786, *SD* = 0.79; *t*_(57.467)_ = 1.017, *p* = 0.313, *d* = 0.263). The average correct recognition rate for task-relevant objects was *M* = 0.908 (*SD* = 0.289), while it was lower for task-irrelevant objects (*M* = 0.626, *SD* = 0.484). With regards to congruence, congruent and incongruent objects were recognized approximately equally likely (congruent: *M* = 0.740, *SD* = 0.439; incongruent: *M* = 0.794, *SD* = 0.404).

Using a logistic mixed effect model with subject-factor as higher level we could unravel a significant main effect of task-relevance on the correct recognition of an object (OR = 0.161, 95% CI [0.097, 0.268]), while there was no significant effect of cortisol group affiliation (OR = 1.467, 95% CI [0.748, 2.874]), congruence (OR = 1.148, 95% CI [0.652, 2.020]) or any tested interaction effect, namely of congruence and task-relevance (OR = 1.533, 95% CI [0.837, 2.810]), of cortisol group affiliation and congruence (OR = 0.855, 95% CI [0.497, 1.470]) or cortisol group affiliation and task-relevance (OR = 0.564, 95% CI [0.306, 1.039]).

Ultimately, if an object was task-relevant or not significantly influenced a correct recognition one day after encoding.

Last but not least, we analyzed the room sortings in cases of false recognitions of lures. Were these were more likely sorted to the semantically fitting room rather than to an unrelated one? Looking at the proportions of sortings across subjects we found that the data was not normal, indicated by Shapiro-Wilk normality test with W = 0.762; p < 0.001 for the proportion of semantically sorted lures and W = 0.762; p < 0.001 for the proportion of unrelatedly sorted lures. Thus, we used Wilcoxon-Signed-Rank-Test and indeed found that falsely recognized lures were more likely sorted to the semantically fitting room rather than to an unrelated room (*V* = 741, *p* < 0.001), showing a semantic bias in false memories.

**Spatial recall**

*Object characteristics*

Within the spatial recall task (SRT), we estimated the spatial distance between the dropped position and the correct position (referred to as *drop error* in the following). On average, task-relevant objects were placed 117.687 unity units (uu, SD = 186.789) away from their correct position, and task-irrelevant objects 282.682 uu (SD = 274.784). Participants had an overall confidence of 4.283 (SD = 1.861) when retrieving the spatial positions of encountered objects.

Replicating the results from our previous study, analyses revealed that task-relevance (*F*_(1, 1428.5)_ = 40.900, *p* < 0.001 , *f^2^* = 0.03), congruence (*F*_(1, 1380.9)_ = 207.278, *p* < 0.001 , *f^2^* = 0.15), and an interaction between these two predictors (*F*_(1, 1380.6)_ = 49.722, *p* < 0.001 , *f^2^* = 0.04) significantly predicted the drop error, that is, task-relevant objects had a lower drop error than task-irrelevant objects, and congruent objects were placed closer to their original position than incongruent objects. Post hoc analyses for the interaction between task-relevance and congruence revealed that there was a significant difference between all pairwise contrasts (all *t*_(>1384)_ >4.623, all *p_Holm_* < 0.001) except for task-relevant congruent and task-irrelevant congruent objects in drop error (*t*_(1420)_ = 0.241, *p_Holm_ = 0.995)*. We additionally included confidence of object placement and cortisol group affiliation as predictors in our model, and found a significant main effect of confidence with a higher confidence resulting in a lower drop error (*F*_(1, 1348.6)_ = 195.976, *p* < 0.001 , *f^2^* = 0.15), but no effect of group affiliation (*F*_(1, 60)_ = 0.233, *p* = 0.233, *f^2^* = 0.004). There was furthermore no interaction between cortisol group affiliation and congruence (*F*_(1, 1380.1)_ = 0.711, *p* = 0.399 , *f^2^* < 0.001) or task-relevance (*F*_(1, 1380.2)_ = 1.602, *p* = 0.206, *f^2^* = 0.001).

Concludingly, the difference in drop error between task-relevant and -irrelevant objects was significant for incongruent objects, but not for congruent objects. Participants who received cortisol instead of placebo did not show a significantly different retrieval pattern overall, but also not when looking at the drop error for congruently or incongruently encountered objects or task-relevant or task-irrelevant objects. Results are summarized in Table S2.

**Table S2** *Pairwise post hoc contrasts of task-relevance and congruence predicting drop error in SRT.*

| contrast | estimate | SE | df | t-ratio | *p*_Holm_ |
| --- | --- | --- | --- | --- | --- |
| Task-relevant congruent – task-irrelevant congruent | 3.96 | 16.5 | 1420 | 0.241 | 0.995 |
| Task-relevant congruent – task-relevant incongruent | 78.09 | 15.1 | 1385 | 5.182 | < 0.001 |
| Task-relevant congruent – task-irrelevant incongruent | 231.68 | 16.5 | 1421 | 14.034 | < 0.001 |
| Task-irrelevant congruent – task-relevant incongruent | 74.13 | 16.0 | 1412 | 4.623 | < 0.001 |
| Task-irrelevant congruent – task-irrelevant incongruent | 227.72 | 15.0 | 1384 | 15.155 | < 0.001 |
| Task-relevant incongruent – task-irrelevant incongruent | 153.59 | 16.1 | 1412 | 9.559 | < 0.001 |

***Note****. Results are averaged over the levels of cortisol group affiliation. Degrees-of-freedom were estimated using Kenward-roger estimation. P-value adjustments were undertaken using Holm-correction for 6 tests.*

*Semantic bias*

We furthermore evaluated whether participants were more likely to place incongruent objects into the semantically fitting room rather than to the unrelated room (i.e.,semantic bias). To this end, we calculated the proportion of room sortings among incongruent objects (‘episodic’, ‘semantic’, ‘unrelated, ‘not sorted’). Note that for this analysis, we filtered all objects which were rated as ‘new’ in the recognition task and treated them as ‘not sorted’, as we cannot be sure that these objects were seen at all in the virtual environment. On average, participants did not recognize 2.596 incongruent objects (min = 1, max = 7), leaving 9.533 objects as the basis for this analysis (min = 5, max = 12). As the data was not normally distributed, we used Wilcoxon Signed Rank Test as non-parametric alternative for a pairwise analysis of systematic differences between proportions within participants. Proportions were estimated separately for task-relevant and task-irrelevant objects. There was no significant difference between the proportion of semantically and unrelatedly sorted task-relevant objects (*V* = 388.5, *p_Holm_* = 0.989), while the proportions significantly differed between semantically and unrelatedly sorted task-irrelevant objects (*V* = 1192.5, *p_Holm_* < 0.001). We conducted the same analysis separately for the cortisol and placebo group, and found the same effect in both groups: while the proportions between semantically sorted objects and unrelatedly sorted objects differed for task-irrelevant objects (cortisol group: V = 285, *p_Holm_* < 0.001, placebo group: *V* = 328.5, *p_Holm_* < 0.05), there was no such difference for task-relevant objects (cortisol group: V = 108, *p_Holm_* = 1, placebo group: *V* = 89.5, *p_Holm_* = 1). Furthermore, we did not find a difference between the proportions of semantically sorted objects between the two groups, neither for task-relevant (V = 403.5, *p_Holm_* = 1), nor for task-irrelevant objects (V = 513.5, *p_Holm_* = 1). To summarize, task-irrelevant objects, which were not sorted to the correct room, were more likely sorted to their semantically fitting rather than to the unrelated room. A coherent visualization is provided in Figure S3.

**Figure S3**

Visualization of room sorting data from spatial recall


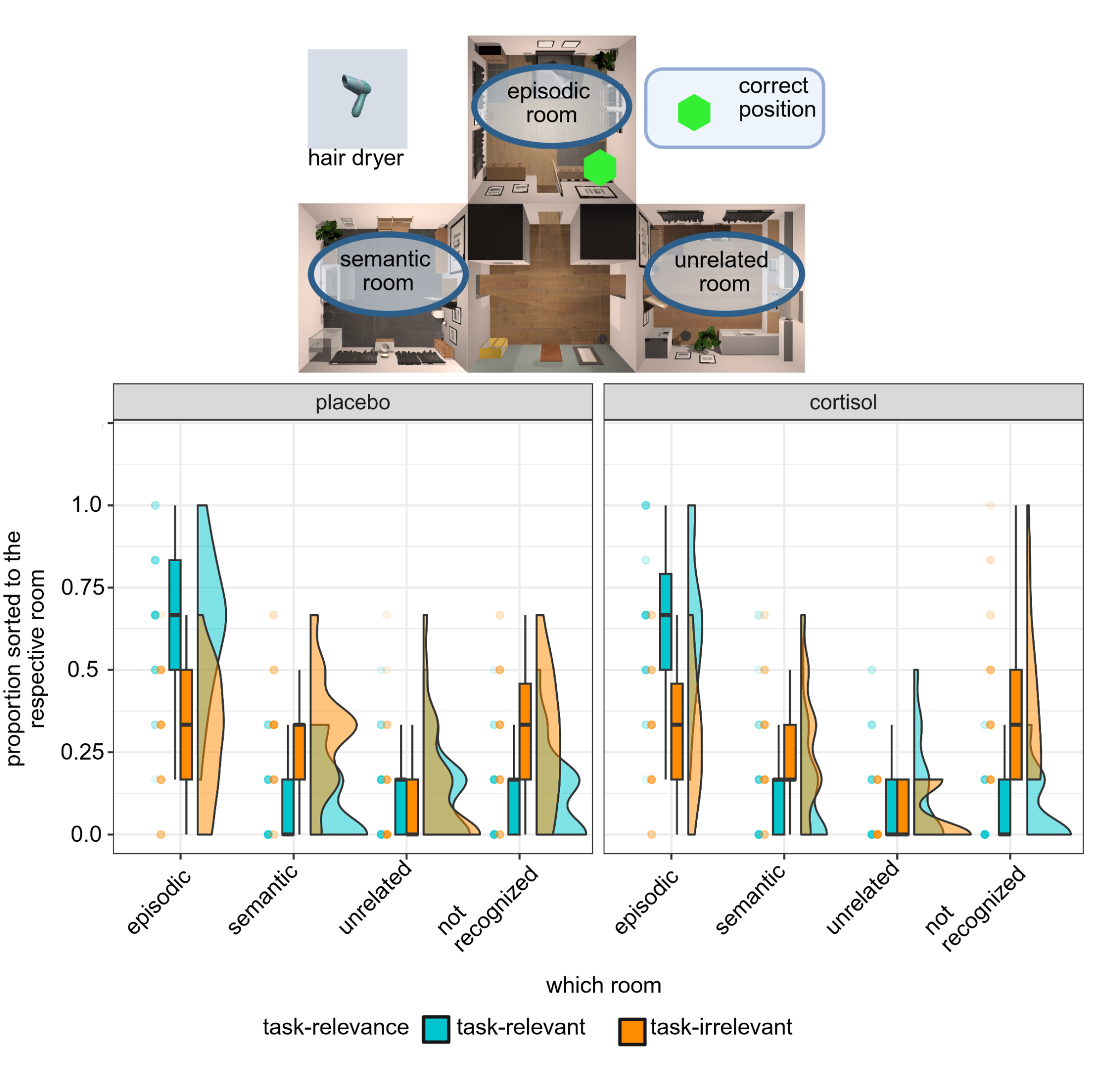


***Note****. Depicted are the distribution of proportions of room sortings of incongruent objects (N = 12). The episodic room refers to the correct room, the semantic room to the semantically associated room, the unrelated room refers to the third room which is neither episodically nor semantically related, and not recognized comprises proportions of objects which were declared as not being recognized from the recognition task.*

**Temporal recall**

*Object characteristics*

We first analyzed whether the distance between two objects in temporal retrieval (including only task-relevant objects and referring to the order of tasks in the episodic virtual encoding task) could be predicted by whether or not they belonged to the same room category or were presented in the same or in different rooms. To control for confabulation through the original distance between the two objects in question, we conducted a separate analysis predicting the distance in the original order of tasks by the same predictors.

Whether two objects were presented in the same room did not significantly predict the retrieval of temporal distance between two objects (*F*_(1, 1918)_ = 0.220, *p_Holm_* = 0.639, *f*^2^ < 0.001). Interestingly however, analyses revealed that tasks with objects belonging to the same semantic room category were retrieved as being temporally closer together compared to objects belonging to different semantic room categories (*F*_(1, 1918)_ = 5.308, *p_Holm_* < 0.05, *f*^2^ = 0.003). This stands in contrast to our finding that the distance between two objects in the original order of tasks was higher for objects belonging to same room category (*F*_(1, 1918)_ = 5.552, *p_Holm_* < 0.05, *f*^2^ = 0.003), as compared to objects belonging to different semantic room categories. We can thus summarize that objects belonging to the same semantic room category seem to be retrieved in closer temporal proximity, even though this effect was opposite in the originally encountered order of tasks. Lastly, we found that tasks of objects being presented in the same room were farther apart than tasks of objects being presented in different rooms (*F*_(1, 1918)_ = 4.018, *p_Holm_* < 0.05, *f*^2^ = 0.003). Note that all effect sizes in this analysis are very small, limiting the interpretability of our findings.

*Semantic bias in temporal recall*

With the aim of estimating semantic bias in temporal recall, just as in the original work presenting this paradigm (Zöllner et al., 2023), we calculated a semantic clustering index for temporal retrieval for each participant using the data derived from the temporal recall task (TRT). That is, we calculated the difference value of the average distance of different room category objects and the average recalled distance of same room category objects, both for the encoded order of tasks and for the retrieved order of tasks. We then used a paired Wilcoxon Signed Rank Test to estimate if the semantic clustering index in retrieval was significantly higher than in the originally presented order of tasks. Indeed, across all participants, we found that the semantic clustering was higher in the retrieved order of tasks as compared to the encoded order of tasks (*V* = 1129.5, *p_Holm_* < 0.05). Interestingly, when estimating this model separately for two groups, we found this main effect to persist in the placebo (*V* = 305, *p_Holm_* < 0.05), but not the cortisol group (*V* = 269.5, *p_Holm_* = 0.133). However, as visible in figure S4, this might be due to a higher semantic clustering index in the encoded order rather than a difference between the two groups at retrieval.

**Figure S4**

*Semantic clustering index, comparing semantic clustering between the original temporal order and the retrieved temporal order during the temporal recall task.*

*
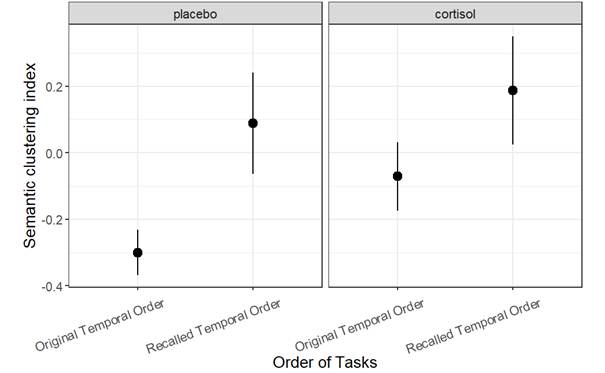
****Note****. The semantic clustering index was calculating by estimating the difference between the average temporal distance between two objects belonging to different semantic room categories and the average temporal distance between two objects belonging to the same semantic room category. This was done separately for the original order of tasks and for the retrieved order of tasks. Depicted are the mean and standard error.*

*Relating gaming experience to memory*

Participants indicated their level of regular gaming experience in a questionnaire. They were either not gaming on regular basis (N = 27 participants), playing monthly (N = 1), weekly (N = 6), several times per week (N = 14) or daily (N = 12).

Exploratively, we related gaming experience to overall memory measures, that is, free recall, indicating how many objects a participant retrieved during free recall, recognition memory in form of deePrime and spatial recall as average drop error. To this end we conducted ANOVAs to estimate differences in memory between the levels of gaming experience. Neither memory measure could be related to differences in gaming experience between the participants (number of objects freely retrieved: F(4, 55) = 0.128, p = 0.972; dee Prime: F(4, 55) = 0.548, p = 0.702; average drop error: F(4, 55) = 0.284, p = 0.887).

**Recall 28 days later**

Participants underwent an additional questionnaire 28 days after encoding. As this questionnaire was taken online, the measures differ from the ones taken one day after encoding. In short, participants were not exposed to a pharmacological manipulation, conducted the tests on their laptops or smartphones, and each test differed from the controlled laboratory setting. The free recall task was conducted via anonymized audio-message, the recognition task was comparable, and the TRT was conducted using numbers participants entered next to each image instead of sorting the objects to the correct position. The SRT was not included. This part of the analysis is exploratory and was preregistered as such.

*Sample*

Seven participants did not fill in the questionnaire and are thus not included in the following analysis. The sample thus consisted of *N* = 53 participants (aged M = 24.19, SD = 4.14), with an average BMI of 24.12 (SD = 2.82) kg/m^2^. *N* = 28 of the participants were part of the cortisol group and *N* = 25 of the placebo group. *N* = 29 participants reported frequent gaming experience, while 24 reported to have had no experience in gaming. *N* = 41 reported to have the highest school-degree (Abitur), *N* = 1 indicated to have a middle-school degree, while *N* = 13 reported to have at a university’s degree (*N* = 1 first law degree, *N* = 7 Bachelor’s degree, 2 Master’s degrees). *N* = 43 had normal vision, while *N* = 10 had corrected-to-normal vision (*N* = 2 with a red-green-weakness).

*Free recall*

On average, each participant freely retrieved *M* = 7.83 objects (*SD* = 3.83), which makes a retrieval rate of 0.333 (SD = 0.16). From those objects, on average *M* = 4.89 (*SD* = 2.79) were retrieved together with a room. *M* = 3.89 objects were recalled in the correct, *M* = 0.42 (*SD* = 0.75) in the semantically fitting and *M* = 0.58 (*SD* = 0.95) in the unrelated, third room.

*Recognition task*

The overall recognition rate across all participants was 87 % (*SD* = 0.12). Dee Prime as a measure of corrected recognition memory was *M* = 1.74 (*SD* = 0.74, as compared to *M =* 1.69, *SD* = 0.83 at day 2).

*Room sorting (subsequent to recognition task)*

With regards to the room sorting, we mirrored the analyses from the room sorting from the SRT. Importantly, unlike during the SRT, participants just indicated the room by selecting it during recognition, not by placing an object spatially into a room.

The data was not normal, we thus used Wilcoxon Signed Rank test for comparisons, and correct for multiple comparisons using Holm-correction. As depicted in figure S5, we found a significant difference between the proportions of objects being sorted to the semantically fitting room and to the unrelated room, for both task-relevant (V = 662.5, *p_Holm_* < 0.001) and task-irrelevant objects (V = 1129.5, *p_Holm_* < 0.001).

**Figure S5**

*Semantic Construction in room recall during the recognition task 28 days after encoding.*

*
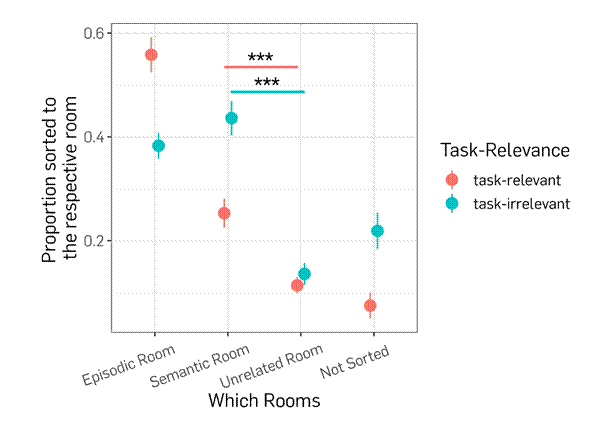
*

***Note****. 28 days after encoding, participants were more likely to sort objects to their semantically fitting rather than to an unrelated room. This was true for task-relevant and -irrelevant objects. Depicted are means and standard errors.*

*Temporal recall*

28 days after encoding, the temporal recall was conducted by depicting all task-relevant objects. Participants were instructed to notate a number from 1 to 12 to each object, referring to the order of tasks they remember to have performed with a specific object.

We again mirrored the analyses from the second day after encoding. First, we thus analyzed whether we find a significant effect of whether two objects belonged to the same room category or not on the recalled temporal distance between two objects (setting subjects as higher level). We thus performed a linear mixed model and set whether two objects were presented in the same room and whether objects belonged to the same semantic room category or not as predictors for the retrieved temporal distance. We found that neither predictor influenced the recalled distance between two objects (same room presented: *F*_(1, 3762)_ = 2.238, *p* = 0.135, *f^2^* < 0.001; same semantic room category: *F*_(1, 3762)_ = 0.212, *p* = 0.645, *f*^2^ < 0.001)

In a next step we calculated the semantic temporal clustering score (average distance between two objects from different semantic categories minus average distance between two objects from the same semantic room category). Again, we used Wilcoxon Signed Rank Test to compare the semantic temporal clustering score during retrieval to the semantic temporal clustering score during encoding. We found no significant difference between the two clustering scores across participants (*V* = 827, *p* = 0.469).

**Immersion**

Descriptively, we looked at responses on the immersion questionnaire, the IPQ. They are included in table S3.

**Table S3.**

Average scores of responses from the IPQ.

|  | All | Cortisol group | Placebo group |
| --- | --- | --- | --- |
| Spatial Presence | 3.52 (1.04) | 3.67 (1.02) | 3.37 (1.05) |
| Involvement | 3.52 (1.12) | 3.41 (0.94) | 3.56 (1.28) |
| Experienced Realism | 2.17 (1.01) | 2.30 (1.06) | 2.05 (0.95) |
| Sense of Being There | 3.47 (1.35) | 3.63 (1.31) | 3.30 (1.38) |

***Note****. Mean (and Standard Deviation) of all four immersion subscales across participants and separately for cortisol and placebo group.*

## S3. Citations and references to packages acquired in Unity Asset Store

**Assets acquired for interior of virtual environment**

- ‘HQ ArchViz Modern Studio Apartment – Version1.2 by NEXT LEVEL 3D (2020)

- ‘Lamp Model” Version 1.2 by HARPETSTUDIO (2019)

- ‘HDRP Furniture Pack” Version 1.0 by TRIDIFY (2019)

- ‘Door Free Pack Aferar” Version 1.0 by ANDREY FERAR (2019)

- ‘ArchVizPRO Interior Vol. 6” Version 1.3 by ARCHVIZPRO (2020)

- ‘Big Furniture Pack” Version 1.3 by VERTEX STUDIO (2017)

- ‘Realistic Furniture and Interior Pack” Version 1.0 by TIRED PUBLISHER (2019)

**Assets acquired as stimuli**

- Clipboard – ‘Clipboard”- Version 1.0 by COOKIEPOPWORKS.COM (2019)

- ‘Sandwiches Sliced”- Version 1.01 by PEACEFIELD (2017)

- ‘Clothing Collection” Version 1.0 by 3D EVERYTHING (2017)

- ‘Cleaning Products” Version 1.0 by 32CM (2019)

- ‘Painless Props – Kitchen” Version 1.2 by LITTLE ARMS STUDIOS (2017)

- ‘Realistic Kitchen Pack!” Version 1.1. by FROGBYTES (2017)

- ‘Painless Props- Bathroom and Laundry” Version 1.3 by LITTLE ARMS STUDIOS (2017)

- ‘Bedroom Asset” Version 1.0 by HARPETS STUDIO (2018)

- ‘Analog Clocks Vol.1” Version 1.0 by ARTPLAYER (2018)

- ‘Super Coffee Pack!” Version 1.0 by FROGBYTES (2018)

- ‘Lamp Model” Version 1.2 by HARPETSTUDIO (2019)

- ‘Japanese food – Sushi Free” Version 1.0 by BRUSHZIM (2019)

- ‘Kitchen Props Free” Version 1.0 by JAKE SULLIVAN (2018)

- Player: ‘Standard Assets (for Unity)” Version 1.1.6 by UNITY TECHNOLOGIES (2020)

**Stimuli created in Blender (**Version 2.83, <https://www.blender.org>)

- Ear plugs
- Sleeping mask

**Assets acquired for stimuli lures for recognition task**

- ‘Fashion shop – interior and exterior” –Version 1.0 by MIXAILL (2020)

- ‘PBR Fruits and Vegetables” – Version 1.1 by DEVDEN (2020)

- ‘Clothing Collection” Version 1.0 by 3D EVERYTHING (2017)

- ‘Cleaning Products” Version 1.0 by 32CM (2019)

- ‘Painless Props – Kitchen” Version 1.2 by LITTLE ARMS STUDIOS (2017)

- ‘Realistic Kitchen Pack!” Version 1.1. by FROGBYTES (2017)

- ‘Painless Props- Bathroom and Laundry” Version 1.3 by LITTLE ARMS STUDIOS (2017)

- ‘Croissants Pack” – Version 1.0 by OPTIMESH (2019)

- ‘Kitchen Props Free” Version 1.0 by JAKE SULLIVAN (2018)

## S4. Citations and references for R-packages

**Table S4**

*References for R-packages used in this publication.*

| ggplot2 | H. Wickham (2016). ggplot2: Elegant Graphics for Data Analysis. Springer-Verlag New York. |
| --- | --- |
| ggpubr | Kassambara A (2023). ggpubr: 'ggplot2' Based Publication Ready Plots. R package version 0.6.0, [https://CRAN.R-project.org/package=ggpubr](https://cran.r-project.org/package=ggpubr). |
| psych | William Revelle (2024). psych: Procedures for Psychological, Psychometric, and Personality Research. Northwestern University, Evanston, Illinois. R package version 2.4.6, [https://CRAN.R-project.org/package=psych](https://cran.r-project.org/package=psych). |
| car | Fox J, Weisberg S (2019). An R Companion to Applied Regression, Third edition. Sage, Thousand Oaks CA.<https://www.john-fox.ca/Companion/>. |
| sjPlot | Lüdecke D (2024). sjPlot: Data Visualization for Statistics in Social Science. R package version 2.8.16, [https://CRAN.R-project.org/package=sjPlot](https://cran.r-project.org/package=sjPlot). |
| lme4 | Bates, D., Mächler, M., Bolker, B., & Walker, S. (2015). Fitting linear mixed-effects models Usinglme4. *Journal of Statistical Software, 67*(1).<https://doi.org/10.18637/jss.v067.i01> |
| lmerTest | Kuznetsova A, Brockhoff PB, Christensen RHB (2017). “lmerTest Package: Tests in Linear Mixed Effects Models.” *Journal of Statistical Software*, *82*(13), 1–26. [doi:10.18637/jss.v082.i13](https://doi.org/10.18637/jss.v082.i13). |
| emmeans | Lenth R (2024). emmeans: Estimated Marginal Means, aka Least-Squares Means. R package version 1.10.5, [https://CRAN.R-project.org/package=emmeans](https://cran.r-project.org/package=emmeans). |
| dplyr | Wickham H, François R, Henry L, Müller K, Vaughan D (2023). dplyr: A Grammar of Data Manipulation. R package version 1.1.4, https://github.com/tidyverse/dplyr,<https://dplyr.tidyverse.org>. |
| tidyr | Wickham H, Vaughan D, Girlich M (2024). tidyr: Tidy Messy Data. R package version 1.3.1, [https://CRAN.R-project.org/package=tidyr](https://cran.r-project.org/package=tidyr). |
| nlme | Pinheiro J, Bates D, R Core Team (2024). nlme: Linear and Nonlinear Mixed Effects Models. R package version 3.1-166, [https://CRAN.R-project.org/package=nlme](https://cran.r-project.org/package=nlme). |
| jtools | Long JA (2022). jtools: Analysis and Presentation of Social Scientific Data. R package version 2.2.0,<https://cran.r-project.org/package=jtools>. |
| afex | Henrik Singmann, Ben Bolker, Jake Westfall and Frederik Aust (2016). *afex: Analysis of Factorial Experiments.* R package version 0.16-1. [https://CRAN.R-project.org/package=afex](https://cran.r-project.org/package=afex) |
| effectsize | Ben-Shachar M, Lüdecke D, Makowski D (2020). effectsize: Estimation of Effect Size Indices and Standardized Parameters. *Journal of Open Source Software, 5*(56), 2815. doi:10.21105/joss.02815 |
| rstatix | Kassambara A (2023). rstatix: Pipe-Friendly Framework for Basic Statistical Tests. R package version 0.7.2,<https://rpkgs.datanovia.com/rstatix/>. |
| ARTool | Kay M, Elkin L, Higgins J, Wobbrock J (2021). *ARTool: Aligned Rank Transform for Nonparametric Factorial ANOVAs*. [doi:10.5281/zenodo.594511](https://doi.org/10.5281/zenodo.594511), R package version 0.11.1,<https://github.com/mjskay/ARTool> |
| ggrain | Allen, M., Poggiali, D., Whitaker, K., Marshall, T. R., van Langen, J., & Kievit, R. A. Raincloud plots: a multi-platform tool for robust data visualization [version 2; peer review: 2 approved] **Wellcome Open Research** 2021, 4:63.<https://doi.org/10.12688/wellcomeopenres.15191.2> |

## S5. Cortisol Analysis

Even though the pharmacological application of cortisol is a reliable method to increase a participants’ hormonal cortisol levels, we checked statistically over all timepoints for group-differences between the cortisol and the placebo group. As the assumption of normality was violated, we log10-transformed cortisol values prior to analysis. The mixed model with subject as higher level and group, time and the interaction of both included as predictors revealed a significant main effect of time (*F*_(3, 162.878)_ = 68.570, *p_Holm_*  < 0.001, *f^2^* = 1.26) and group (*F*(_1, 56.485)_ = 215.642, *p_Holm_* < 0.001, *f^2^* = 3.82), and furthermore a significant time x group interaction effect (*F*_(3, 162.878)_ = 94.488, *p_Holm_* < 0.001, *f^2^* = 1.74). With regards to main effects, post hoc pairwise comparisons showed a significantly higher cortisol level for the cortisol as compared to the placebo group (*t*_(57.9)_ = 14.683, *p* < 0.001), a significant difference in cortisol levels between baseline and all other timepoints (all t > 7.670, all *p_Holm_* < 0.001) and +30min. and all following timepoints (all t > 6.506, all *p_Holm_* < 0.001). Furthermore, we found no group difference at baseline (*t*_(159)_ = 0.698, *p_Holm_* = 1), but a significant difference at +30 min. (*t*_(164)_ = 16.258, *p*_Holm_ < 0.001), +90 min. (*t*_(158)_ = 14.035, *p*_Holm_ < 0.001) and +120 min. (*t*_(165)_ > 12.843, *p*_Holm_ < 0.001). While cortisol levels increased over time in the cortisol group (baseline to all subsequent timepoints: all *t*_(164)_ > 13.703, all *p_Holm_* < 0.001), we found a decrease in cortisol levels in the placebo group between baseline and +90 min. (*t*_(163)_ = 3.461, *p*_Holm_ < 0.01), baseline and +120 min. (*t*_(163)_ = 3.256, *p*_Holm_ < 0.05), +30 and +90 min (*t*_(163)_ = 2.836, *p*_Holm_ < 0.05), reflecting the circadian rhythm.

As a control measure, we acquired subjective stress from participants in form of negative affect. As the assumption of normality was violated, we performed an ART (Aligned Rank Transformed) -ANOVA. We found that there was no significant difference between the two groups in negative affect (*F*_(1, 57.977)_ = 2.673, *p* = 0.107, *f^2^* = 0.05), but a significant main effect of time (*F*_(3, 163.071)_ = 3.481, *p* < 0.05, *f^2^* = 0.06). The data yielded no significant interaction effect of group and time (*F*_(3, 163.071)_ = 1.712, *p* = 0.167, *f^2^* = 0.03). Post hoc comparisons showed a significant decrease in negative affect across groups between baseline and +90 min. (*t*_(163)_ = 3.143, *p*_Holm_ < 0.05), but no other significant difference between the other timepoints acquired.

The results are depicted In figure S6.

**Figure S6.**

**Salivary cortisol levels and negative affect over time**

**
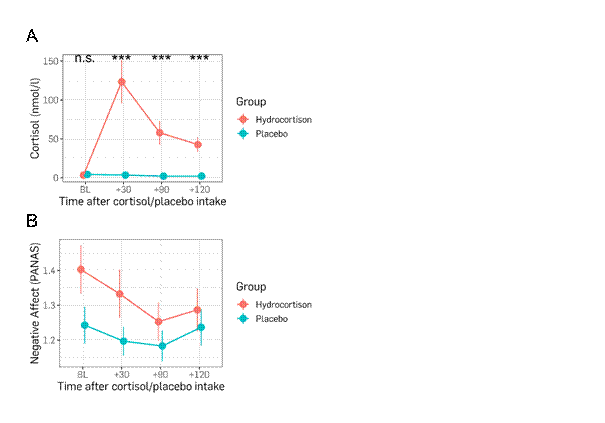
**

***Note****. Depicted are the respective mean values and standard errors. A. Group differences between the cortisol and placebo group in cortisol levels (nmol/l) over time. At all time points but at baseline, cortisol levels of the cortisol receiving group were significantly higher than the placebo group. Over time, cortisol increased significantly in the cortisol receiving, but not in the placebo group. B. Group differences between the cortisol and placebo group in Negative Affect, measured with PANAS questionnaire, over time. There were no significant group differences.*

## S6. Statistical results RSA: pre-encoding

In the following, we provide an exhaustive overview of model results, summarized in tables. We used linear mixed models, which were tested for significance using Wald III ANOVA. Before being reported in the manuscript, p-values were corrected for multiple comparisons using Holm-correction.

*Pre-encoding similarity*

**Table S5.**

**A-D reference to the different ROIs (VVS, LOC, lingual gyrus, hippocampus) for which the analyses of pattern similarity pre-encoding predicted by category affiliation were performed.**

**A)**

**
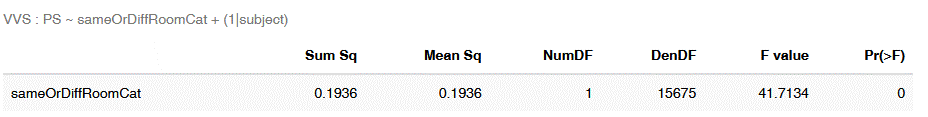
**

**B)**

**
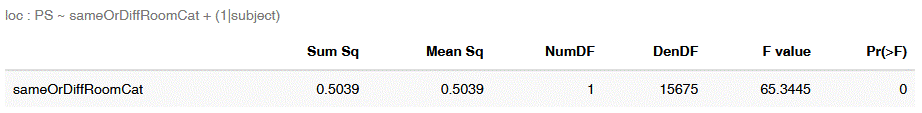
**

**C)**

**
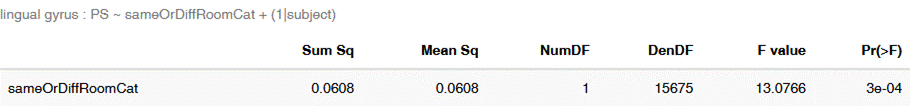
**

**D)**

**
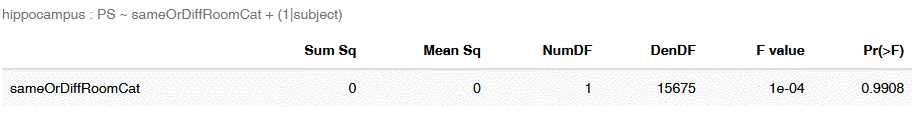
**

## S7. Statistical results RSA: post-encoding, mean similarity of incongruent objects to their semantically fitting congruent counterparts

In the following, we provide an exhaustive overview of model results, summarized in tables. Note that in order to investigate group differences with regards to all our predictors, group affiliation was included in all tested models and is thus reported repeatedly. We used mixed anovas for within- and between-subject measures. Before inclusion in the manuscript, p-values were corrected for multiple comparisons using Holm-correction.

**Task-relevance**

**Table S6.**

*A-D reference to the different ROIs (anterior hippocampus, posterior hippocampus, LOC and lingual gyrus) for which the analyses of mean pattern similarity of incongruent objects to their semantically fitting congruent counterparts predicted by task-relevance was conducted.*

**A)**

**
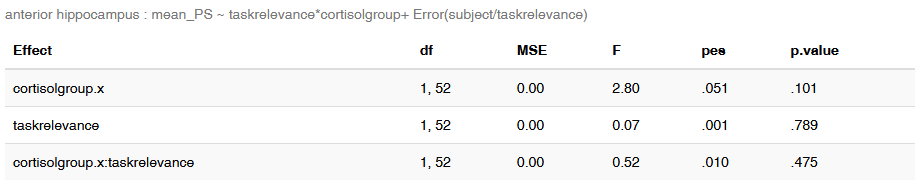
**

**B)**

**
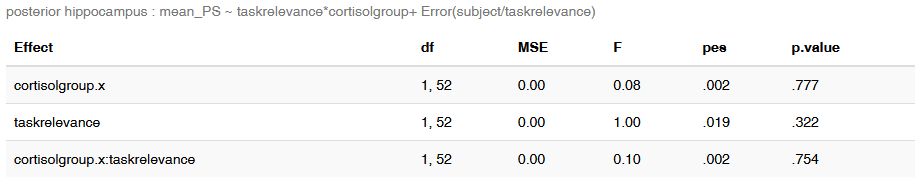
**

**C)**

**
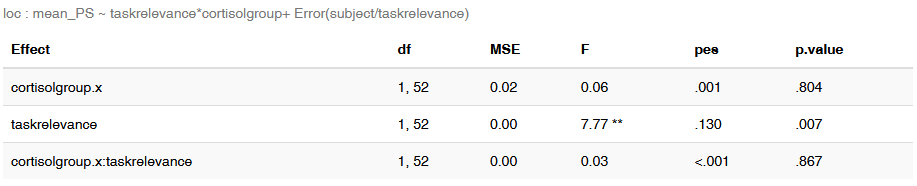
**

**D)**

**
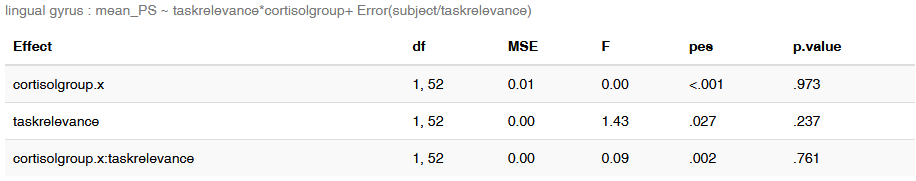
**

**Room-placement**

How many items are included in mean values for A semantically and B correctly sorted objects per participant?

**Figure S7**

**A)**

**semantic placements**

**
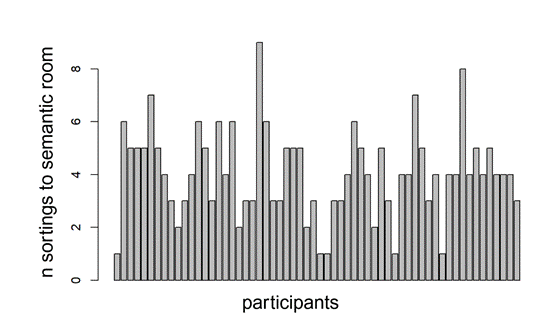
**

**B)**

**correct placements**

***
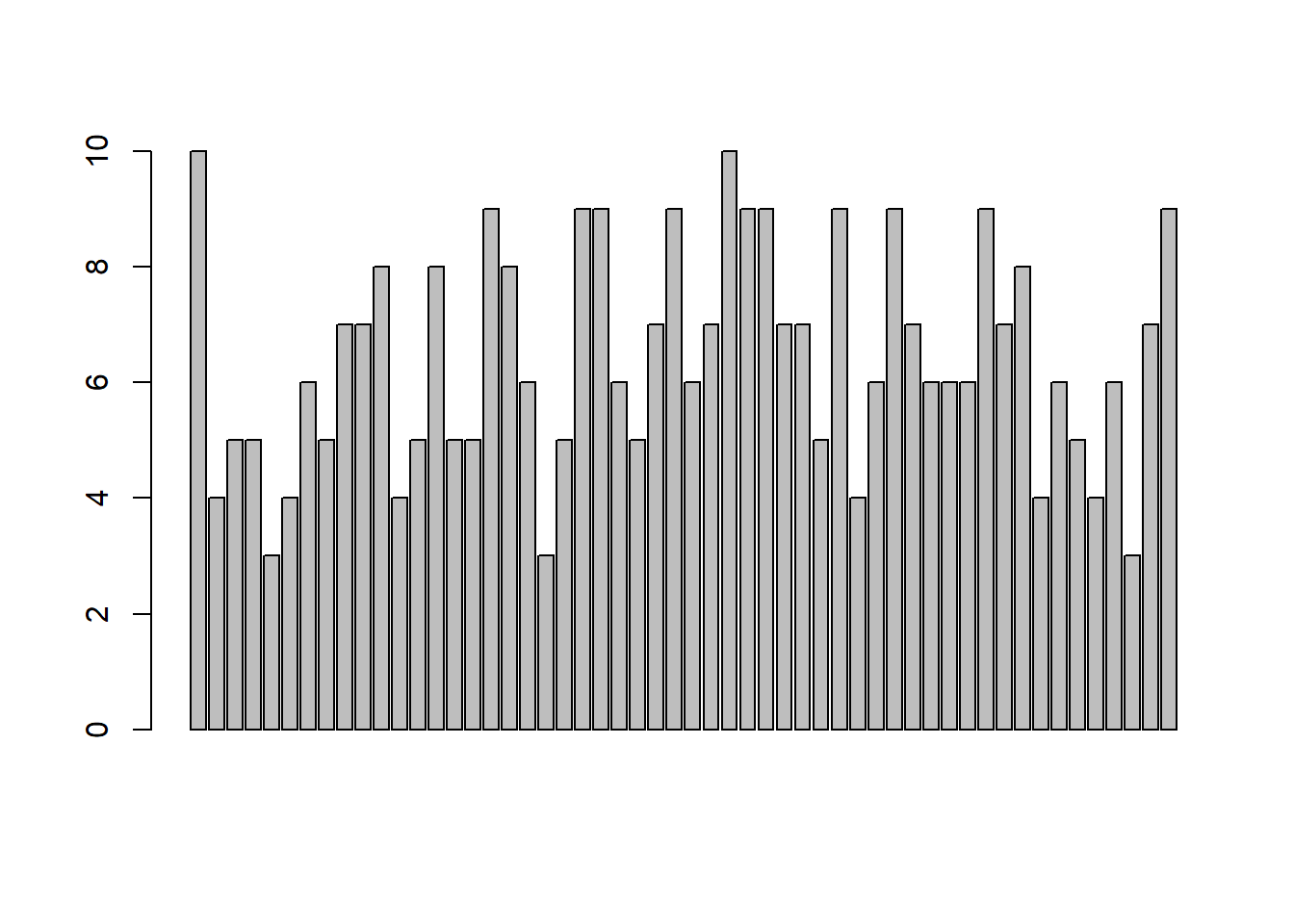
***

***Note****. For each participant (X-axis), the number of semantically sorted objects is indicated (y-axis). This refers to room-sortings during spatial retrieval.*

**Table S7.**

*A-D reference to the different ROIs (anterior hippocampus, posterior hippocampus, LOC and lingual gyrus) for which the analyses of mean pattern similarity of incongruent objects to their semantically fitting congruent counterparts predicted by which room they were sorted to, the correct, episodic one or the semantically fitting room.*

**A)**

**
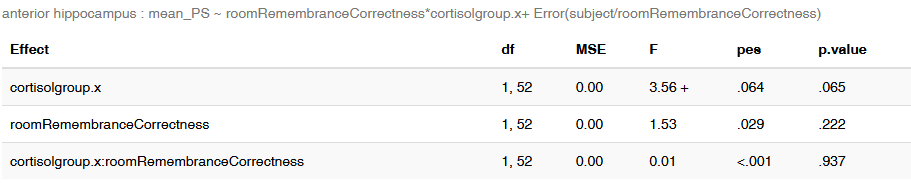
**

**B)**

**
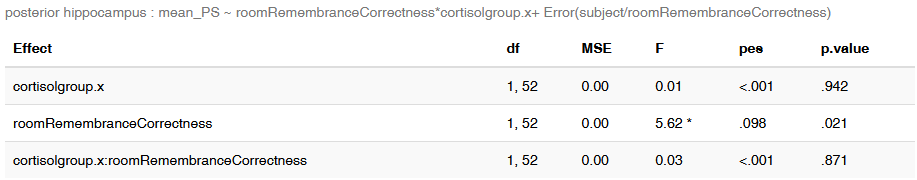
**

**C)**

**
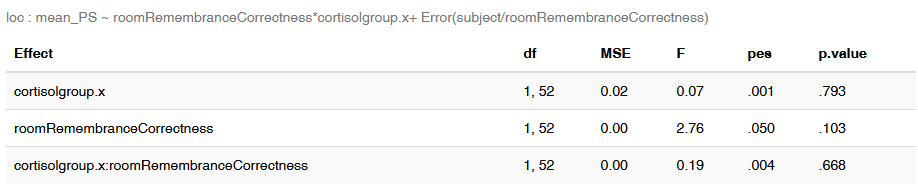
**

**D)**

**
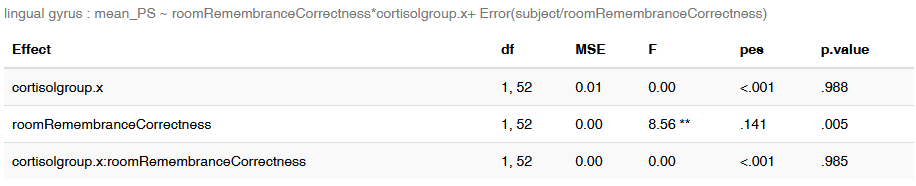
**

## S8. Statistical results RSA: PS-change (post minus pre encoding)

In the following, we provide an exhaustive overview of model results, summarized in tables. Note that in order to investigate group differences with regards to all our predictors, group affiliation was included in all tested models and is thus reported repeatedly. We used linear mixed models, which were tested for significance using Wald III ANOVA. Note that group affiliation was added as a predictor to all models to estimate main- and interaction effects. Before inclusion in the manuscript, p-values were corrected for multiple comparisons using Holm-correction.

**Remembered in same or different room during spatial recall task**

**Table S8.**

*A-D reference to the different ROIs (anterior hippocampus, posterior hippocampus, LOC and lingual gyrus) for which the analyses of pattern similarity change predicted by whether participants retrieved the object pair in the same semantic room and the interaction with group affiliation were performed.*

**A)**

**
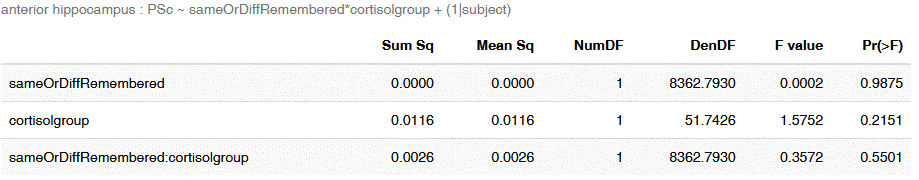
**

**B)**

**
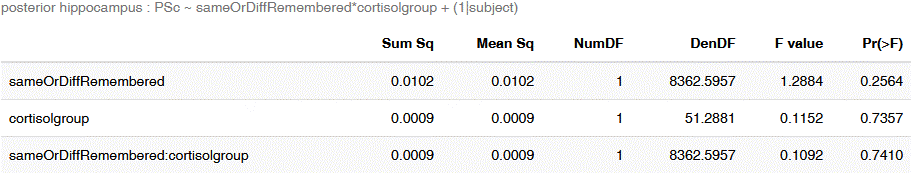
**

**C)**

**
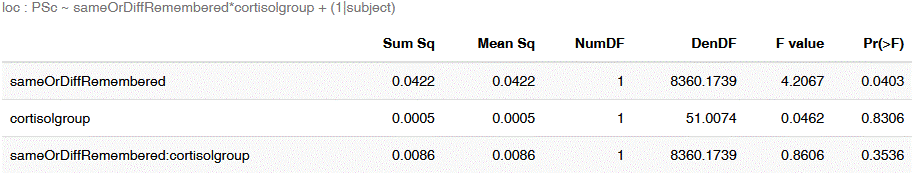
**

**D)**

**
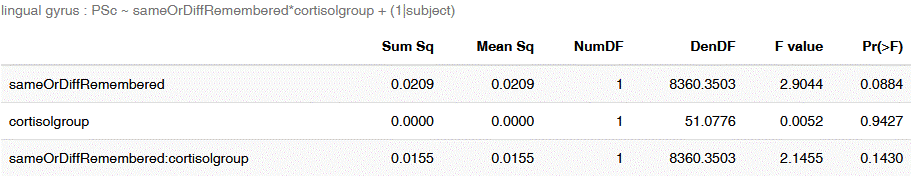
**

**Distance between two objects during spatial recall task**

**Table S9.**

*A-D reference to the different ROIs (anterior hippocampus, posterior hippocampus, LOC and lingual gyrus) for which the analyses of pattern similarity change predicted by whether participants retrieved the object pair in the same semantic room and the interaction with group affiliation were performed.*

**A)**

**
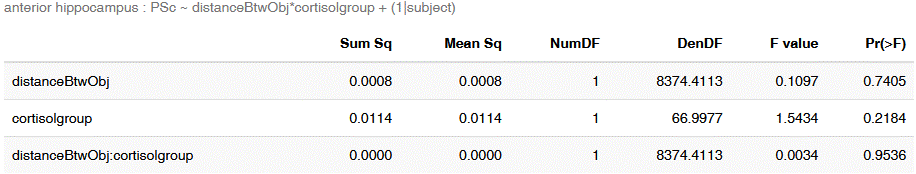
**

**B)**

**
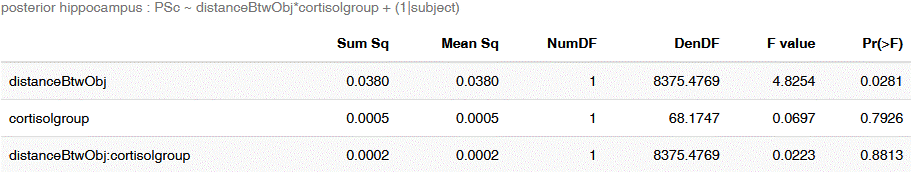
**

**C)**

**
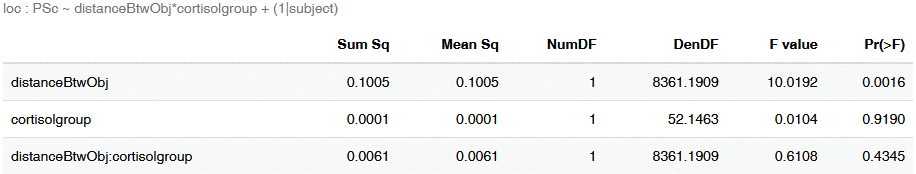
**

**D)**

**
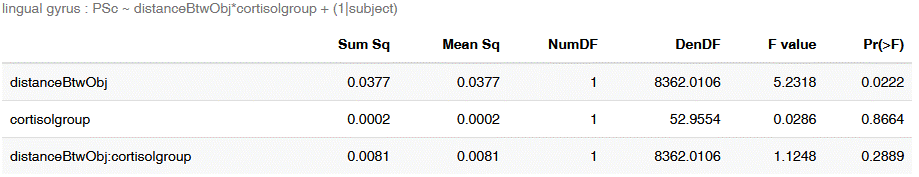
**

**Confidence (average confidence of spatial retrieval)**

**Table S10.**

*A-D reference to the different ROIs (anterior hippocampus, posterior hippocampus, LOC and lingual gyrus) for which the analyses of pattern similarity change predicted by how confident participants were with regards to the spatial retrieval of the object pair and the interaction with group affiliation were performed.*

**A)**

**
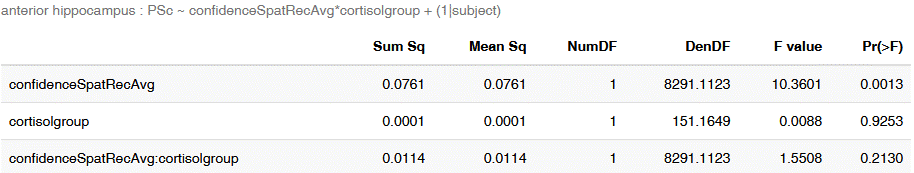
**

**B)**

**
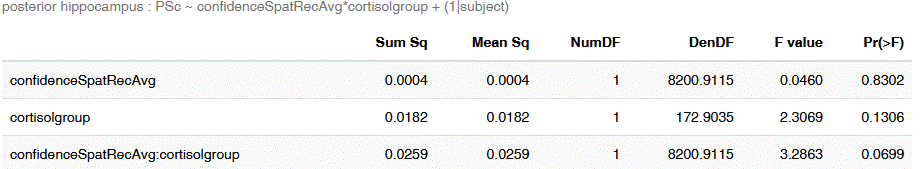
**

**C)**

**
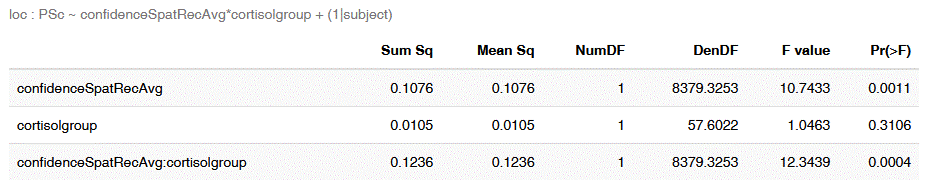
**

**D)**

**
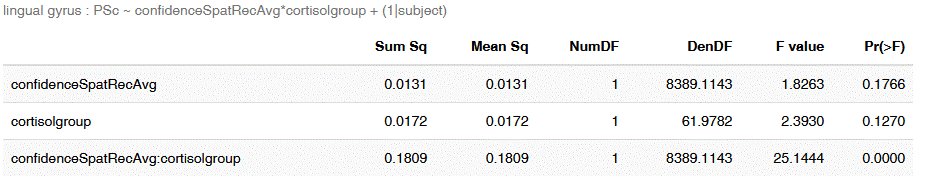
**

## S9. Statistical results RSA: pattern reorganization

In the following, we provide an exhaustive overview of model results, summarized in tables. Note that in order to investigate group differences with regards to all our predictors, group affiliation was included in all tested models to estimate interaction effects and is thus reported repeatedly. We used linear mixed models, which were tested for significance using Wald III ANOVA. Before inclusion in the manuscript, p-values were corrected for multiple comparisons using Holm-correction.

**Accuracy (Correct room)**

**Table S11.**

*A-D reference to the different ROIs (anterior hippocampus, posterior hippocampus, LOC and lingual gyrus) for which the analyses of encoding-retrieval pattern similarity of an object predicted by whether it was retrieved correctly or not and the interaction with group affiliation.*

**A)**

**
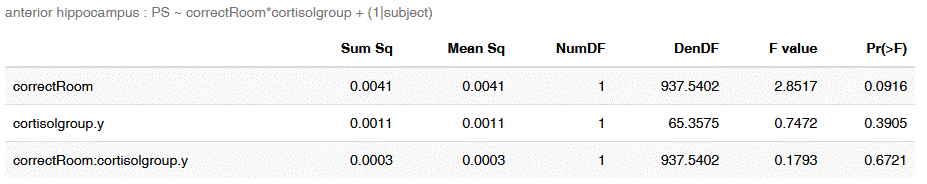
**

**B)**
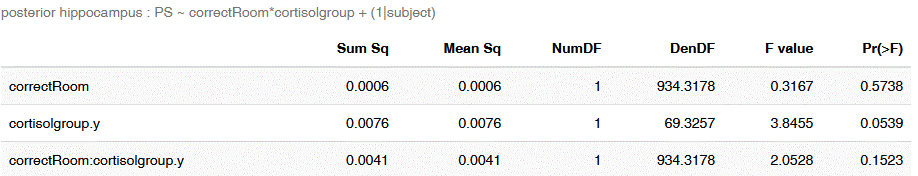


**C)**

**
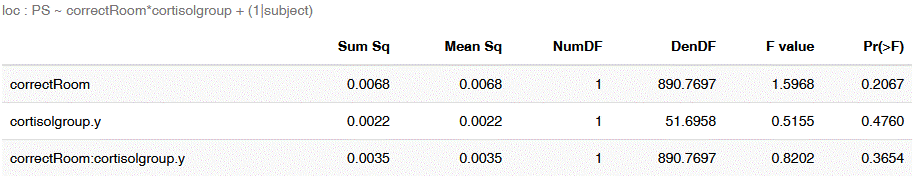
**

**D)**

**
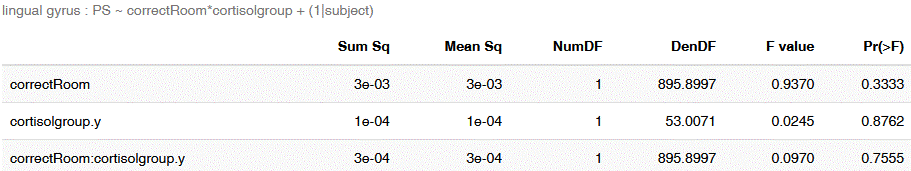
**

**Accuracy (Drop error)**

**Table S12.**

*A-D reference to the different ROIs (anterior hippocampus, posterior hippocampus, LOC and lingual gyrus) for which the analyses of encoding-retrieval pattern similarity of an object predicted by how far it was placed away from its correct position and the interaction with group affiliation.*

**A)**

**
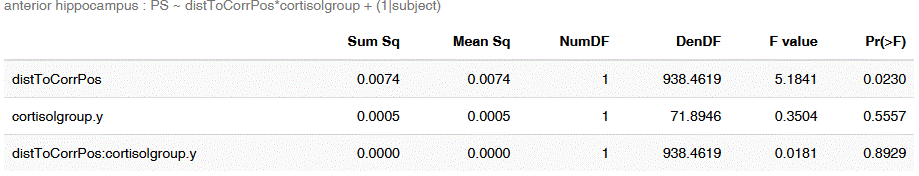
**

**B)**

**
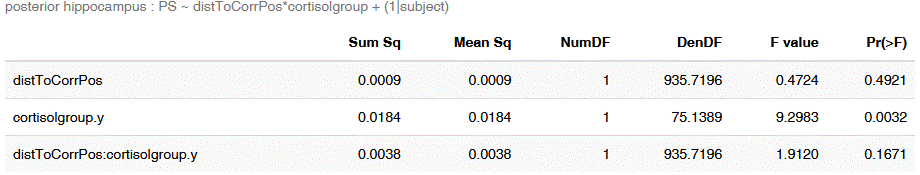
**

**C)**

**
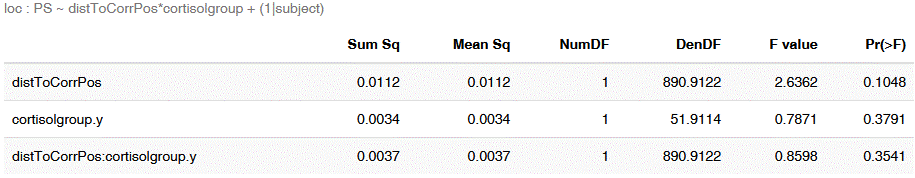
**

**D)**

**
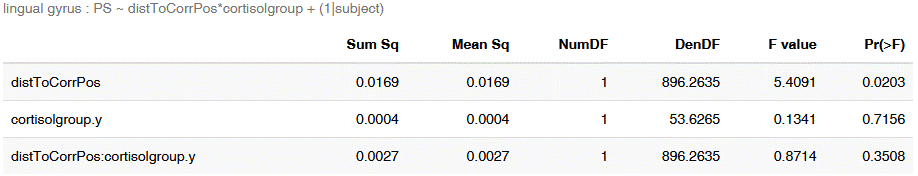
**

**Confidence**

**Table S13.**

*A-D reference to the different ROIs (anterior hippocampus, posterior hippocampus, LOC and lingual gyrus) for which the analyses of encoding-retrieval pattern similarity of an object predicted by how confident a participant was when spatially retrieving this object, and the interaction with group affiliation.*

**A)**

**
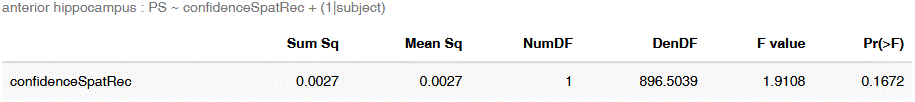
**

**B)**

**
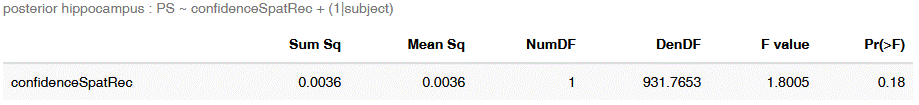
**

**C)**

**
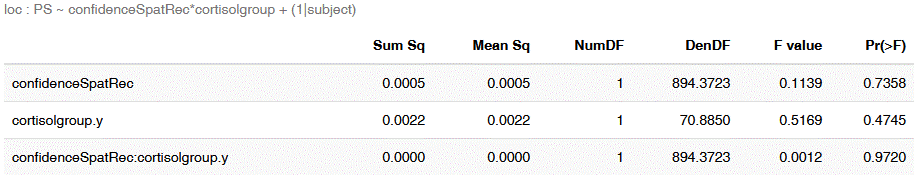
**

**D)**

**
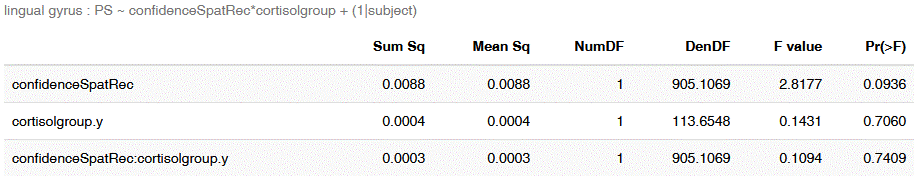
**

**Congruence**

**Table S14.**

*A-D reference to the different ROIs (anterior hippocampus, posterior hippocampus, LOC and lingual gyrus) for which the analyses of encoding-retrieval pattern similarity of an object predicted by whether it was encountered congruently or incongruently and the interaction with group affiliation.*

**A)**

**
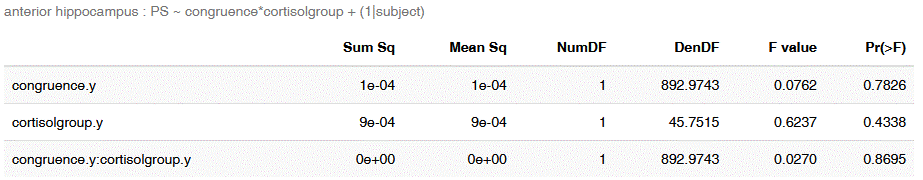
**

**B)**

**
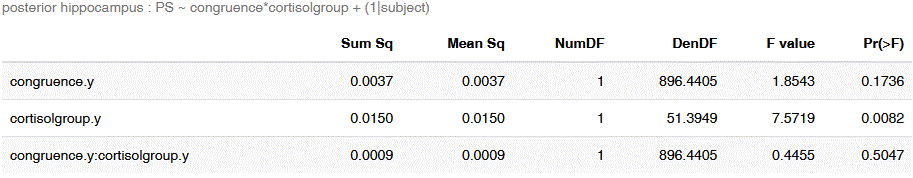
**

**C)**

**
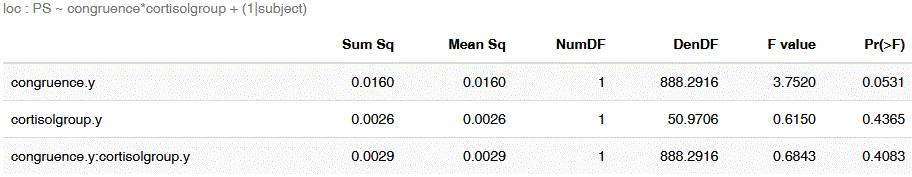
**

**D)**

**
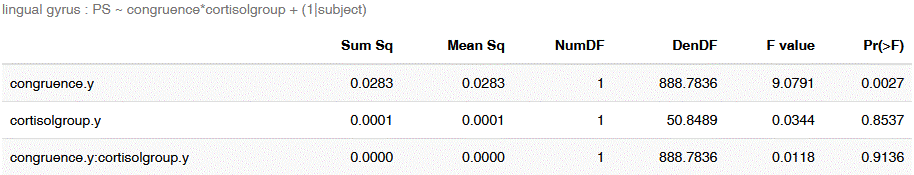
**

**Task-relevance**

**Table S15.**

*A-D reference to the different ROIs (anterior hippocampus, posterior hippocampus, LOC and lingual gyrus) for which the analyses of encoding-retrieval pattern similarity of an object predicted by whether it was interacted with (task-relevant) or not (task-irrelevant) and the interaction with group affiliation.*

**A)**

**
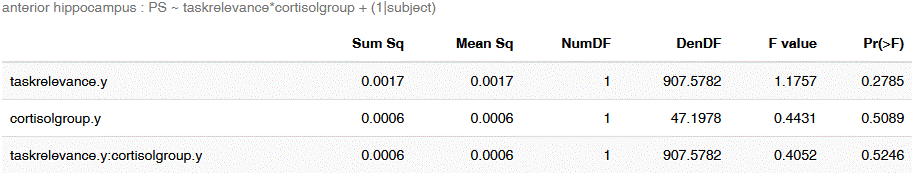
**

**B)**

**
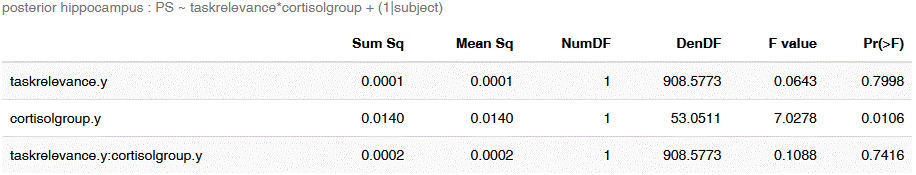
**

**C)**

**
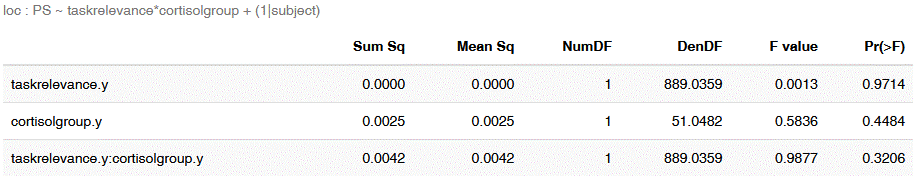
**

**D)**

**
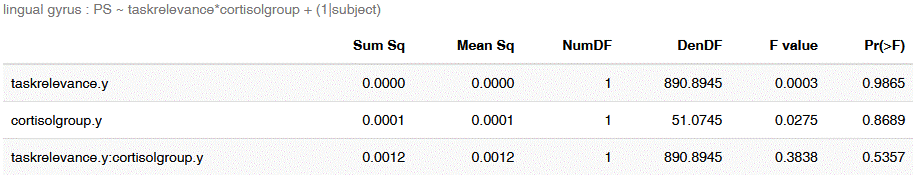
**

## S10. Analysis of drop error in pattern reorganization

There was no significant effect of correct room placement on pattern reorganization of an object in any ROI included in our analyses (aHC, pHC, LOC and lingual, all *p_Holm_* > 0.1). However, when looking at the distance between the retrieved location and the correct location (drop error), we found a trend towards a significant main effect in the aHC (*F*_(1, 892.32)_ = 5.184, *p* < 0.05, *p_Holm_* = 0.081, *f^2^* = 0.009), and the lingual gyrus (*F*_(1, 896.26)_ = 5.409, *p* < 0.05, *p_Holm_* = 0.081, *f^2^* = 0.006, see figure S8B), however not surviving corrections for multiple comparisons. That is, a lower drop error (i.e., higher accuracy in spatial recall) seems to slightly be associated with a higher pattern reorganization. Again, the interaction between drop error and group affiliation did not result in significant effects. This might support findings for reinstatement effects on memory recall. This is in line with previous findings on neural pattern reinstatement and supports that the gist of the episode has been encoded and consolidated (Bird et al., 2015; Tompary & Davachi, 2017) resulting in better memory performance.

**Figure S8**

*Pattern reorganization predicted by drop-error as a measure of memory accuracy*


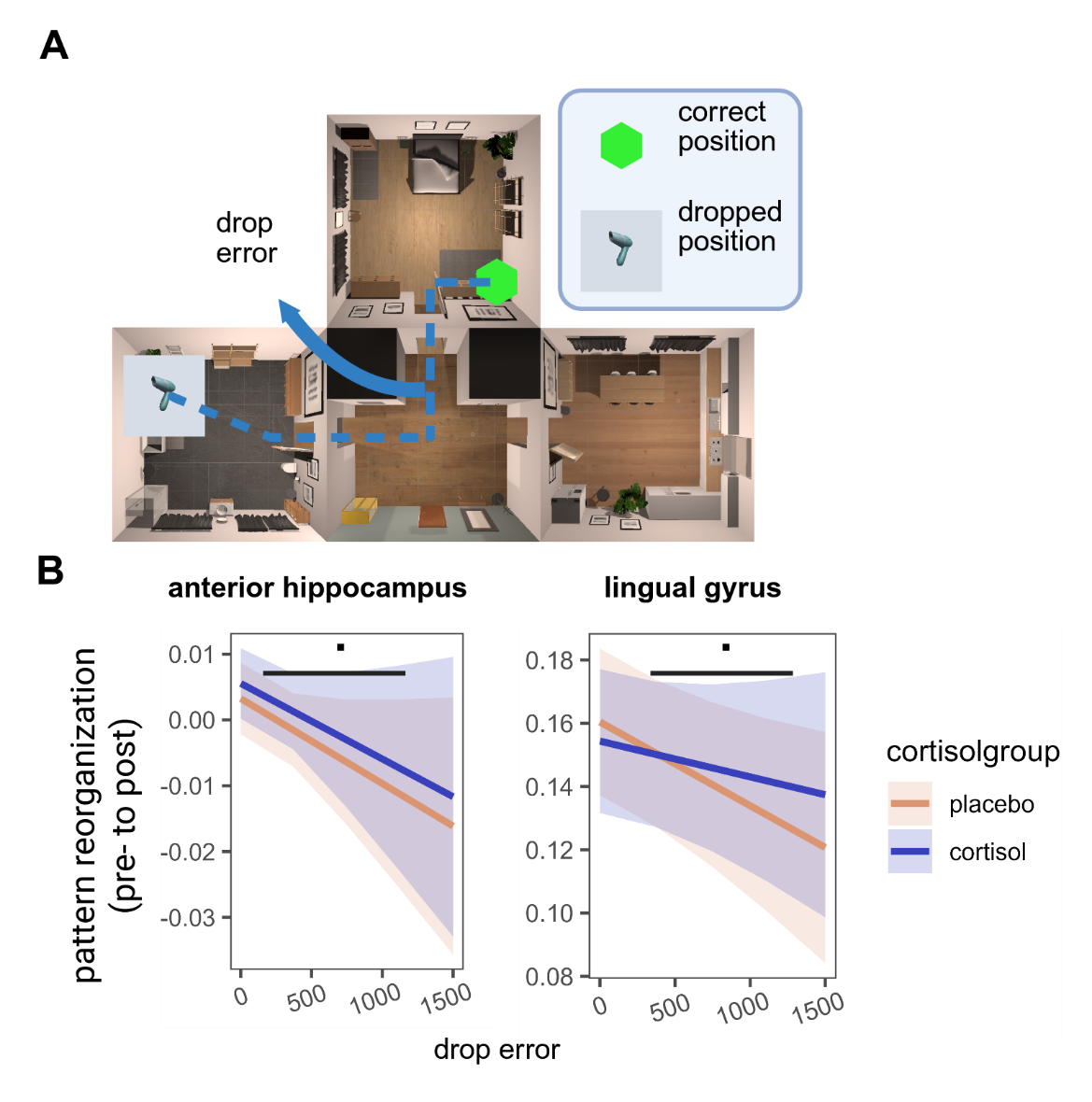


***Note*.** *A. visualization of how drop error was calculated based on spatial object retrieval. B. we observed a trend-significance pre-correction in anterior hippocampus and lingual gyrus, showing that a higher drop error was associated with a decrease in pre- post-encoding similarity.*

Publication bibliography

Bird, C. M., Keidel, J. L., Ing, L. P., Horner, A. J. & Burgess, N. (2015). Consolidation of Complex Events via Reinstatement in Posterior Cingulate Cortex. *The Journal of neuroscience : the official journal of the Society for Neuroscience*, *35*(43), 14426–14434. https://doi.org/10.1523/JNEUROSCI.1774-15.2015

Brainard, David H. (1997): The Psychophysics Toolbox. In *Spatial Vis* 10 (4), pp. 433–436. DOI: 10.1163/156856897x00357.

Breyer, B.; Bluemke, M. (2016): Deutsche Version der Positive and Negative Affect Schedule PANAS (GESIS Panel).

Otto, Tobias; Rose, Jonas (2023): The open toolbox for behavioral research. In *Behavior research methods*. DOI: 10.3758/s13428-023-02199-x.

Schubert, Thomas; Friedmann, Frank; Regenbrecht, Holger (2001): The Experience of Presence: Factor Analytic Insights. In *Presence: Teleoperators & Virtual Environments* 10 (3), pp. 266–281. DOI: 10.1162/105474601300343603.

Tompary, A. & Davachi, L. (2017). Consolidation Promotes the Emergence of Representational Overlap in the Hippocampus and Medial Prefrontal Cortex. *Neuron*, *96*(1), 228-241.e5. https://doi.org/10.1016/j.neuron.2017.09.005

Zöllner Carina; Klein, Nicole; Cheng, Sen; Schubotz, Ricarda I.; Axmacher, Nikolai; Wolf, Oliver T. (2023): Where was the toaster? A systematic investigation of semantic construction in a new virtual episodic memory paradigm. In *Quarterly journal of experimental psychology (2006)* 76 (7), pp. 1497–1514. DOI: 10.1177/17470218221116610.
